# Regret Bound for Multi-Armed Bandits with Discounting: A Robust Stochastic Control Approach

**DOI:** 10.1016/j.fmre.2025.10.003

**Published:** 2025-10-15

**Authors:** Zhou Zhou

**Affiliations:** School of Mathematics and Statistics, University of Sydney, Sydney 2006, Australia

**Keywords:** Multi-armed bandits with discounting, Regret bound, Robust stochastic control, Bayesian optimization, Entropy regularization

## Abstract

We investigate the exact regret bound for stochastic multi-armed bandit problems with discounting, by formulating the regret bound as the value of a robust stochastic control problem. We first show that the regret bound coincides with the value associated with the Bayesian optimization problem under the worst prior. However, a solution to this Bayesian optimization may not be an optimal strategy for the robust control problem. To resolve this dilemma, we introduce an entropy regularization term into the cost function. In this way, we prove the existence of a saddle point for the entropy-regularized robust control. Moreover, the unique optimal strategy for the Bayesian optimization under the worst prior is also an (almost) optimal strategy for the original robust stochastic control problem.

## 1 Introduction

In a basic multi-armed bandit (MAB) problem, an agent selects an arm among K arms (i.e., make a decision from K options) at each time. The agent receives the reward of the selected arm, but cannot observe the outcome of other arms. The distribution of each arm is unknown to the agent, and the agent could learn the distribution by observing the outcomes of selected arms. The agent aims to find a best strategy to maximize the cumulative reward or minimize the regret, and thus face the tradeoff between exploration and exploitation. MAB problems have been investigated extensively in many variations in the literature, and have a lot of applications in different fields. We refer to the book [1] and the references therein.

A popular research direction for MAB is to design certain algorithms and analyse their regret bound asymptotically in terms of the number of rounds T and the number of arms K. The regret bound (e.g., $O(\sqrt{KT\ln(T)})$) typically is only in the asymptotic sense, and it is not clear what would be the exact bound (e.g., not clear what is the constant term in $O(\sqrt{KT\ln(T)}$, and it could be very large). The same applies to the lower bound in the worst case scenario (e.g., $O(\sqrt{KT})$) for the regret. In addition, such analysis for the regret bound cannot be readily applied to the case when the MAB problem admits discounting.

To analyze the exact (not asymptotic) regret bound and to handle the case with discounting, we investigate the regret bound in the worst case scenario using a robust stochastic control approach. Specifically, we consider the problem

$$\underset{\pi }{\inf}\underset{\theta \in \Theta }{\sup}V\left(\theta ,\pi \right)\;\text{with}\;V\left(\theta ,\pi \right):=E^{\theta ,\pi }\left[\sum_{t=0}^{T}\delta^{t}\left(X_{t}\left(a^{*}\left(\theta \right)\right)-X_{t}\left(A_{t}\right)\right)\right].$$

where π is a relaxed control strategy, θ represents a model for the joint distribution of K arms, $X_{t}\left(k\right)$ is the outcome of the k-th arm at time t, $a^{*}\left(\theta \right)$ represents the optimal strategy if the true model θ is known, $A_{t}$ is the arm selected at time t under strategy π, δ is a discount factor, and T∈N or T=∞ is the time horizon. The set Θ represents the collection of admissible models, which could include all the distributions on a given space or some classes of distributions parametrized by θ. Here V(θ,π) is interpreted as the regret bound with strategy π given that the true but unknown model is θ. Then $inf_{\pi }sup_{\theta \in \Theta }V\left(\theta ,\pi \right)$ represents the exact optimal regret bound in the worst case scenario. Let us emphasize that in our problem each model θ is static and does not evolve over time. That is, we consider stochastic MAB instead of adversary MAB. From a machine learning point of view, this makes learning more effective, as the agent could observe the outcomes of selected arms and make a better guess accordingly for the prefixed true model. From a stochastic control point of view, θ being static prevents us from directly applying the dynamic programming principle to solve the problem.

As our first main result (Theorem 2.1), we prove that

(1) $$\begin{array}{ccc} \underset{\pi }{\inf}\underset{\theta \in \Theta }{\sup}V\left(\theta ,\pi \right) & = & \underset{\pi }{\inf}\underset{q\in P(\Theta )}{\sup}V\left(q,\pi \right)=\underset{q\in P(\Theta )}{\sup}\underset{\pi }{\inf}V\left(q,\pi \right)\\ & = & \underset{q\in P(\Theta )}{\sup}\underset{\alpha }{\inf}V\left(q,\alpha \right), \end{array}$$

where P(Θ) is the set of probability measures on Θ, α is a pure strategy, and with a bit of abuse of notation, $V\left(q,\pi \right):=\int_{\Theta }V\left(\theta ,\pi \right)q\left(d\theta \right)$. When the state space for $X_{t}$ is finite, we further show that there exists a saddle point $\left(q^{*},\pi^{*}\right)$ for V. Note each q∈P(Θ) is a mixture of θ∈Θ and can be thought of as a prior distribution for models. For each q∈P(Θ), the problem $inf_{\alpha }V\left(\theta ,\alpha \right)$ is a dynamic Bayesian optimization. Then (1) indicates that the exact regret bound coincides with the value associated with the Bayesian optimization under the worst prior. In addition, the problem $\tilde{V}\left(q\right):=inf_{\alpha }V\left(q,\alpha \right)=inf_{\pi }V\left(q,\pi \right)$ can be solved using the dynamic programming principle. It is worth noting that the values $\left\{\tilde{V}\left(q\right),\;q\in P\left(\Theta \right)\right\}$ together are related by a dynamic programming equation, and thus in theory can be obtained simultaneously.

On the other hand, our first main result may not help in finding the optimal relaxed strategy $\pi^{*}\in arg\;min_{\pi }sup_{\theta \in \Theta }V\left(\theta ,\pi \right)$. Specifically, suppose we have obtained the worst prior $q^{*}\in \text{arg}\max_{q\in P(\Theta )}\tilde{V}\left(q\right)$ and an optimal strategy $\hat{\pi }\in arg\;min_{\pi }V\left(q^{*},\pi \right)$ (or $\hat{\alpha }\in arg\;min_{\alpha }V\left(q^{*},\alpha \right)$) for $\tilde{V}\left(q^{*}\right)$ from the dynamic programming equation. There is no guarantee that this $\hat{\pi }$ (or $\hat{\alpha }$) would be an optimal strategy for $inf_{\pi }sup_{\theta \in \Theta }V\left(\theta ,\pi \right)$ (see Remark 2.8). To resolve this dilemma, we modify the cost function V by introducing an entropy regularization term,

$$V^{\eta ,\lambda }\left(q,\pi \right):=V\left(q,\pi \right)-\eta \psi \left(q\right)-\lambda \int_{\Theta }E^{\theta ,\pi }\left[\sum_{t=0}^{T}\delta^{t}\Gamma \left(\pi_{t}\right)\right]q\left(d\theta \right),$$

where η,λ>0, ψ(·) is a strictly convex function on P(Θ), and Γ is Shannon’s entropy

$$\Gamma \left(u\right):=-\sum_{k=1}^{K}u_{k}\ln\left(u_{k}\right),\;u=\left(u_{1},\cdots ,u_{k}\right).$$

As our second main result (Theorem 2.2), we show that there exists a unique saddle point $\left(q^{*},\pi^{*}\right)$ for $V^{\eta ,\lambda }$, and $\pi^{*}$ is the unique optimizer for the problem $inf_{\pi }V^{\eta ,\lambda }\left(q^{*},\pi \right)$. As a result, we may find the robust optimal strategy $\pi^{*}\in arg\;min_{\pi }sup_{q\in P(\Theta )}V^{\eta ,\lambda }\left(q,\pi \right)$ by solving the dynamic programming equation for the value function ${\tilde{V}}^{\eta ,\lambda }\left(q\right):=inf_{\pi }V^{\eta ,\lambda }\left(q,\pi \right),\;q\in P\left(\Theta \right)$, and extracting the unique maximum $q^{*}\in \text{arg}\max_{q\in P(\Theta )}{\tilde{V}}^{\eta ,\lambda }\left(q\right)$ and the unique optimizer $\pi^{*}\in arg\;min_{\pi }V^{\eta ,\lambda }\left(q^{*},\pi \right)$. When η,λ>0 are small, $\pi^{*}$ turns out to be an approximate optimizer for the original problem $inf_{\pi }sup_{\theta \in \Theta }V\left(\theta ,\pi \right)$ (see Corollary 2.1). Our result also provides a mathematical justification, from a robust control point of view, for the involvement of entropy regularization in reinforcement learning and stochastic control (see e.g., [2], [3] and the references therein).

We apply the minimax theorem to show (1) and Kakutani-Fan’s fixed-point theorem to prove the existence of a saddle point. A technical difficulty for the proof of (1) lies in the nonconvexity of the map π↦V(q,π) (see Remark 3.1 and Example 3.1). We work on the law of ${\left(A_{t}\right)}_{t}$ conditioned on the path of ${\left(X_{t}\right)}_{t}$, instead of π, in order to recover the convexity (linearity). Another challenge, specifically for the proof of Theorem 2.2, is the noncompactness of the relaxed control set. To overcome this difficulty, we establish the uniform continuity of optimal relaxed control strategies under certain assumptions for models θ∈Θ. This enables us to apply the Arzela-Ascoli theorem and focus on a compact subset of the control set. A key step in the proof of our results is to show the continuity of the map $\left(q,\pi \right)\mapsto V^{\eta ,\lambda }\left(q,\pi \right)$, which is not straightforward.

Our results contribute to the literature of reinforcement learning including the analysis of regret bound for MAB problems. In most cases regret bounds obtained in the literature are in the asymptotic sense, and cannot deal with the situation when discounting is presented. Here we consider the exact bound under the case with discounting using a stochastic control approach. We provide a method to compute the exact bound and obtain an (almost) optimal strategy, by converting the robust control problem to a Bayesian optimization that can be solved using the dynamic programming principle. Admittedly, when the set of models Θ is infinite, the dynamic programming equation for $\tilde{V}\left(q\right)$ (and ${\tilde{V}}^{\eta ,\lambda }\left(q\right)$), q∈P(Θ), would be infinite dimensional, and how to numerically solve such equation remains to be investigated. We leave it for future research.

Our paper also contributes to the research of stochastic control. There have been a lot of works on stochastic control under model uncertainty, see [4], [5], [6], [7], [8], to name a few. In these papers, an underlying model can be time-varying and the set of models is closed under pasting. Consequently, (up to some technicalities) the dynamic programming principle can be directly applied. In our case, since each model θ is static and thus the set of models over multiple time periods is not closed under pasting. As a result, dynamic programming cannot be directly applied. Such difficulty is overcome with the equivalence between the robust control and Bayesian optimization, as is shown in the paper. Our results also indicate that involving an entropy term is helpful in finding an optimal control strategy. This provides a justification, from a robust control point of view, for the entropy regularization in many stochastic control and optimal stopping problems considered these days (see [2], [3], [9], [10], [11], among others). Let us also mention the works [12], [13], [14] where a different framework, called adaptive robust control, is used to deal with model uncertainty.

The rest of the paper is organized as follows. In the next section, we provide the setup and main results. In Section 3, we present the proofs for results from Section 2. The remainder of this section is devoted to frequently used notation, which will also be introduced when it first appears later in the paper.

• N:={0,1,⋯} and K:={1,2,⋯,K}.
• $\Omega^{A}:=K\times K\times \cdots$ $\Omega^{X}:=R^{K}\times R^{K}\times \cdots$, and $\Omega :=\Omega^{A}\times \Omega^{X}$.
• $B(\Omega^{X})$ is the collection of Borel sets of $\Omega^{X}$. Similar definition applies to $\Omega^{A}$ and Ω.
• For $a=\left(a_{0},a_{1},\cdots \right)\in \Omega^{A}$ and $x=\left(x_{0},x_{1},\cdots \right)=({\left(x_{0}\left(k\right)\right)}_{k\in K},$ ${\left(x_{1}\left(k\right)\right)}_{k\in K},\cdots )\in \Omega^{X}$, denote $a_{t}:=\left(a_{0},\cdots ,a_{t}\right)$, $x_{t}:=\left(x_{0},\cdots ,x_{t}\right)$ and $x_{t}\left(a_{t}\right):=\left(x_{0}\left(a_{0}\right),\cdots ,x_{t}\left(a_{t}\right)\right)$.
• $A={\left(A_{t}\right)}_{t\in N}$ and $X={\left(X_{t}\right)}_{t\in N}$ are processes on Ω, such that for $\left(a,x\right)\in \Omega =\Omega^{X}\times \Omega^{A}$,
  $$A\left(a,x\right)=a,\;A_{t}\left(a,x\right)=a_{t},\;X\left(a,x\right)=x,\;X_{t}\left(a,x\right)=x_{t},\;X_{t}\left(k\right)\left(a,x\right)=x_{t}\left(k\right).$$
  Denote $A_{t}:=\left(A_{0},\cdots ,A_{t}\right)$, $X_{t}:=\left(X_{0},\cdots ,X_{t}\right)$ and $X_{t}\left(a_{t}\right):=\left(X_{0}\left(a_{0}\right),\cdots ,X_{t}\left(a_{t}\right)\right)$.
• $H_{t}:=K^{t+1}\times R^{t+1}$ and $H_{t}:=\left(A_{0},\cdots ,A_{t},X_{0}\left(A_{0}\right),\cdots ,X_{t}\left(A_{t}\right)\right)$.
• $\Delta :=\left\{\left(u_{1},\cdots ,u_{k}\right)\in {\left[0,1\right]}^{K}:\;\sum_{k=1}^{K}u_{k}=1\right\}$.
• P(Θ) is the set of probability measures on (Θ,B(Θ)).
• For $u=(u_{1},\cdots ,u_{k})\in \Delta$, define the Shannon’s entropy $\Gamma \left(u\right):=-\sum_{k\in K}u_{k}\ln\left(u_{k}\right)$.
• Denote
  $$A:=\{\alpha ={\left(\alpha_{t}\right)}_{t\in N}:\;\alpha_{t}:H_{t-1}\mapsto K\;\text{is}\;\text{Borel}\;\text{measurable}\}$$
  the set of pure strategies. Denote
  $$\begin{array}{ccc} A^{r}: & = & \{\pi ={\left(\pi_{t}\right)}_{t\in N}:\;\pi_{t}=\left(\pi_{t}\left(1\right),\cdots ,\pi_{t}\left(K\right)\right):\;H_{t-1}\mapsto \Delta \\ & & \text{is}\;\text{Borel}\;\text{measurable}\} \end{array}$$
  the set of relaxed strategies.
• For a probability measure $p^{\theta }$ on $\left(R^{K},B\left(R^{K}\right)\right)$, denote $P^{\theta }:=p^{\theta }\times p^{\theta }\times \cdots$ the probability measure on $\left(\Omega^{X},B\left(\Omega^{X}\right)\right)$. Denote $p_{k}^{\theta }$ the marginal distribution of $p^{\theta }$ for the k-th component. For a transitional kernel $P^{\pi }$ from $\left(\Omega^{X},B\left(\Omega^{X}\right)\right)$ to $\left(\Omega^{A},B\left(\Omega^{A}\right)\right)$, denote $P^{\theta ,\pi }:=P^{\theta }⊗P^{\pi }$. Denote $E^{\theta ,\pi }\left[\cdot \right]$ (resp. $E^{\theta }\left[\cdot \right]$) the expectation under $P^{\theta ,\pi }$ (resp. $P^{\theta }$).

## 2 Setup and main results

In this section, we provide the setup and main results (Theorems 2.1 and 2.2) of this paper. The proofs for results in this section are postponed to Section 3.

Denote N:={0,1,⋯}. Let K∈N with K≥2, representing the number of arms. Denote K:={1,2,⋯,K}. Let $\Omega^{A}:=K\times K\times \cdots$ (resp. $\Omega^{X}:=R^{K}\times R^{K}\times \cdots$), representing the space for the indices of chosen arms (resp. space for the outcomes for the K arms) for all the time steps. Denote $\Omega :=\Omega^{A}\times \Omega^{X}$. For $x=\left(x_{0},x_{1},\cdots \right)=\left({\left(x_{0}\left(k\right)\right)}_{k\in K},{\left(x_{1}\left(k\right)\right)}_{k\in K},\cdots \right),x^{\prime }=\left(x_{0}^{\prime },x_{1}^{\prime },\cdots \right)=\left({\left(x_{0}^{\prime }\left(k\right)\right)}_{k\in K},{\left(x_{1}^{\prime }\left(k\right)\right)}_{k\in K},\cdots \right)\in \Omega^{X}$, define the distance between x and $x^{\prime }$

$$d_{X}\left(x,x^{\prime }\right)=\sum_{t=0}^{\infty }\frac{1}{2^{t}}\sum_{k=1}^{K}\left|x_{t}\left(k\right)-x_{t}^{\prime }\left(k\right)\right|,$$

so that $\Omega^{X}$ is a compete separable metric space under $d_{X}$. We can similarly define the metric on $\Omega^{A}$. Denote $B(\Omega^{X})$ the set of Borel subsets of $\Omega^{X}$. Similar definition applies to $\Omega^{A}$ and Ω. Let $A={\left(A_{t}\right)}_{t\in N}$ and $X={\left(X_{t}\right)}_{t\in N}$ be processes on Ω, such that for

$$\begin{array}{ccc} \omega & = & \left(a,x\right)=\left(a_{0},a_{1},\cdots ,x_{0},x_{1},\cdots \right)\\ & = & (a_{0},a_{1},\cdots ,{\left(x_{0}\left(k\right)\right)}_{k\in K},{\left(x_{1}\left(k\right)\right)}_{k\in K},\cdots )\in \Omega , \end{array}$$

we have

$$\begin{array}{ccc} A(a,x) & = & a,\;A_{t}\left(a,x\right)=a_{t},\;X\left(a,x\right)=x,\;X_{t}\left(a,x\right)\\ & = & x_{t},\;X_{t}\left(k\right)\left(a,x\right)=x_{t}\left(k\right). \end{array}$$

We will also view A and X as canonical processes on $\Omega^{A}$ and $\Omega^{X}$ respectively. Denote $H_{t}:=K^{t+1}\times R^{t+1}$, and

$$H_{t}:=\left(A_{0},\cdots ,A_{t},X_{0}\left(A_{0}\right),\cdots ,X_{t}\left(A_{t}\right)\right).$$

Here each $h_{t}=\left(a_{0},\cdots ,a_{t},z_{0},\cdots ,z_{t}\right)\in H_{t}$ is interpreted as the observed information up to time t, indicating that at time i arm $a_{i}$ is selected and $z_{i}$ is its realized outcome, for i=0,⋯,t. A strategy for selecting an arm at each time will be adapted to the observed history.

Definition 2.1 $\alpha ={\left(\alpha_{t}\right)}_{t\in N}$ is said to be a pure strategy, if for each t∈N,

$$\alpha_{t}:\;H_{t-1}\mapsto K\;\text{is}\;\text{Borel}\;\text{measurable}.$$

Denote A the set of pure strategies. $\pi ={\left(\pi_{t}\right)}_{t\in N}$ is said to be a relaxed strategy, if for each t∈N,

$$\pi_{t}=\left(\pi_{t}\left(1\right),\cdots ,\pi_{t}\left(K\right)\right):\;H_{t-1}\mapsto \Delta \;\text{is}\;\text{Borel}\;\text{measurable},$$

where

(2) $$\Delta :=\left\{\left(u_{1},\cdots ,u_{k}\right)\in {\left[0,1\right]}^{K}:\;\sum_{k=1}^{K}u_{k}=1\right\}.$$

Denote $A^{r}$ the set of relaxed strategies. We use the notation $\pi_{t}\left(h_{t-1}\right)=\left(\pi_{t}\left(1\right)\left(h_{t-1}\right),\cdots ,\pi_{t}\left(k\right)\left(h_{t-1}\right)\right)$ for $h_{t-1}\in H_{t-1}$.

Let Θ be a polish space. For each θ∈Θ, let $p^{\theta }$ be a probability measure on $R^{K}$, representing a possible model (joint distribution) for the rewards of K arms at each trial. Throughout the paper, all the related probability measures are endowed with the weak topology, unless otherwise specified or is clear from the context. We assume $\theta \mapsto p^{\theta }$ is Borel measurable. For θ∈Θ, denote

(3) $$P^{\theta }:=p^{\theta }\times p^{\theta }\times \cdots$$

the probability measure on $\left(\Omega^{X},B\left(\Omega^{X}\right)\right)$. For $\pi ={\left(\pi_{t}\right)}_{t\in N}\in A^{r}$, denote $P^{\pi }$ the transition kernel from $\left(\Omega^{X},B\left(\Omega^{X}\right)\right)$ to $\left(\Omega^{A},B\left(\Omega^{A}\right)\right)$ such that for $x=\left({\left(x_{0}\left(k\right)\right)}_{k\in K},{\left(x_{1}\left(k\right)\right)}_{k\in K},\cdots \right)\in \Omega^{X}$,

(4) $$\begin{array}{ccc} P_{x}^{\pi }\left(A_{t}=k\;\right|\;A_{0} & = & a_{0},\cdots ,A_{t-1}a_{t-1})\\ & = & =\pi_{t}\left(k\right)\left(a_{0},\cdots ,a_{t-1},x_{0}\left(a_{0}\right),\cdots ,x_{t-1}\left(a_{t-1}\right)\right). \end{array}$$

Denote

$$P^{\theta ,\pi }:=P^{\theta }⊗P^{\pi }$$

the probability measure on (Ω,B(Ω)). Here (3) says that under each model θ, $X_{0},X_{1},\cdots$ are i.i.d.. The condition (4) means that the distribution of arms being selected at time t depends on the arms that are selected in previous time steps, as well as the realization of those selected arms, i.e., depends on $h_{t-1}=\left(a_{0},\cdots ,a_{t-1},x_{0}\left(a_{0}\right),\cdots ,x_{t-1}\left(a_{t-1}\right)\right)\in H_{t-1}$. Such distribution is independent of the realization of arms that are not selected previously, and independent of the realization of the arms in the future.

For k∈K and θ∈Θ, denote $p_{k}^{\theta }$ the marginal distribution of $p^{\theta }$ for the k-th component. For each k∈K, we assume there is a probability measure $p_{k}^{*}$ on (R,B(R)) such that $p_{k}^{\theta }\ll p_{k}^{*}$ for any θ∈Θ. Denote $f_{k}^{\theta }:=\frac{d\;p_{k}^{\theta }}{d\;p_{k}^{*}}$ the Radon-Nikodym derivative for k∈K and θ∈Θ. We make the following assumption.

Assumption 2.1 There exists a constant $\tilde{C}\in \left(0,\infty \right)$ such that

(5) $$E^{p_{k}^{*}}\left|X_{0}\left(k\right)\right|\leq \tilde{C},\;\text{and}\;f_{k}^{\theta }\leq \tilde{C},\;p_{k}^{*}\text{-a.s.},\;\forall \;k\in \Theta ,\;\theta \in \Theta .$$

Moreover, $f_{k}^{\theta }$ is continuous in θ, i.e.,

$$\lim_{n\to \infty }f_{k}^{\theta_{n}}=f_{k}^{\theta },\;p_{k}^{*}\text{-a.s.},\;\forall \;\theta_{n},\theta \in \Theta \;\text{with}\;\theta_{n}\to \theta .$$

Example 2.1 Assume X is finitely supported, i.e., for each k∈K there is a finite set $S_{k}\subset R$ such that

$$p_{k}^{\theta }\left(S_{k}\right)=1,\;\forall \;\theta \in \Theta .$$

Then (5) is satisfied, for which we can take $p_{k}^{*}$ as the uniform distribution on $S_{k}$.

Let either T∈N or T=∞. Let δ∈(0,1] be a discount factor, and we require δ<1 when T=∞. We consider the following robust optimization problem, which defines the exact optimal regret bound for a multi-armed bandit problem under discounting.

(6) $$R:=\underset{\pi \in A^{r}}{\inf}\underset{\theta \in \Theta }{\sup}E^{\theta ,\pi }\left[\sum_{t=0}^{T}\delta^{t}\left(X_{t}\left(a^{*}\left(\theta \right)\right)-X_{t}\left(A_{t}\right)\right)\right],$$

where $E^{\theta ,\pi }$ (resp. $E^{\theta }\left[\cdot \right]$) represents the expectation under the probability measure $P^{\theta ,\pi }$ (resp. $P^{\theta }$), and $a^{*}\left(\theta \right)$ is the optimal strategy given the model θ is fully known, i.e.,

$$a^{*}\left(\theta \right)\in \text{arg}\max_{k\in K}E^{\theta }\left[X_{0}\left(k\right)\right].$$

Remark 2.1 Obviously, A can be thought of as a subset $A^{r}$, with $\alpha_{t}\left(h_{t-1}\right)=k$ being treated as $\alpha_{t}\left(h_{t-1}\right)=\left(0,0,\cdots ,0,1,0\cdots ,0\right)$, the k-th component being 1, for α∈A. As a result, the probability measure $P^{\theta ,\alpha }$ can be defined on (Ω,B(Ω)) in a nature way. On the other hand, given a trajectory of X, the trajectory of A would be uniquely determined under a pure strategy. Hence, we may define the probability measure $P^{\theta ,\alpha }$ on $\left(\Omega^{X},B\left(\Omega^{X}\right)\right)$.

Remark 2.2 One could also consider the problem with only pure strategies involved. That is,

$$\hat{R}:=\underset{\alpha \in A}{\inf}\underset{\theta \in \Theta }{\sup}E^{\theta ,\alpha }\left[\sum_{t=0}^{T}\delta^{t}\left(X_{t}\left(a^{*}\left(\theta \right)\right)-X_{t}\left(A_{t}\right)\right)\right].$$

However, this may lead to a larger regret bound in general. Consider an example where we have two coins. The probabilities to get a head (and thus receive $1) for each toss are 1/3 and 2/3 for these two coins, but we do not know which coin has the higher probability. That is, in this example K={1,2},Θ={1,2}, and $X_{0}\left(k\right)∼\text{Bernoulli}\;\left(2/3\right)$ if k=θ, and $X_{0}\left(k\right)∼\text{Bernoulli}\;\left(1/3\right)$ if k≠θ. Consider the one-period case T=0. We can easily get that R=1/6 and $\hat{R}=1/3$.

Remark 2.3 In (6) the true model θ∈Θ does not change over time and does not dynamically react to π, i.e., we consider stochastic MAB. This is very different from the adversarial MAB case, e.g., in [1, chapter 9]. θ being static prevents us from directly applying dynamic programming principle to solve the problem (6).

### 2.1 First main result

Denote P(Θ) the set of probability measures on (Θ,B(Θ)). For q∈P(Θ) and $\pi \in A^{r}$, denote

$$V\left(q,\pi \right):=\int_{\Theta }E^{\theta ,\pi }\left[\sum_{t=0}^{T}\delta^{t}\left(X_{t}\left(a^{*}\left(\theta \right)\right)-X_{t}\left(A_{t}\right)\right)\right]q\left(d\theta \right).$$

With a bit of abuse of notation, set $V\left(\theta ,\pi \right):=V\left(\delta_{\theta },\pi \right)$ for θ∈Θ. For α∈A, we also use the notation V(q,α) and V(θ,α) as α can be regarded as an element in $A^{r}$ by remark Remark 2.1. Below is our first main result.

Theorem 2.1 *Let* Assumption 2.1 *hold. If either* Θ *is compact or* X *is finitely supported (i.e., under the setting of* Example 2.1*), then*

(7) $$\begin{array}{ccc} \underset{\pi \in A^{r}}{\inf}\underset{\theta \in \Theta }{\sup}V\left(\theta ,\pi \right) & = & \underset{\pi \in A^{r}}{\inf}\underset{q\in P(\Theta )}{\sup}V\left(q,\pi \right)\\ & = & \underset{q\in P(\Theta )}{\sup}\underset{\pi \in A^{r}}{\inf}V\left(q,\pi \right)=\underset{q\in P(\Theta )}{\sup}\underset{\alpha \in A}{\inf}V\left(q,\alpha \right) \end{array}$$

*If we assume both* Θ *is compact and* X *is finitely supported, then there exists a saddle point* $\left(q^{*},\pi^{*}\right)\in P\left(\Theta \right)\times A^{r}$*, i.e.,*

$$V\left(q,\pi^{*}\right)\leq V\left(q^{*},\pi^{*}\right)\leq V\left(q^{*},\pi \right),\;\forall \;\left(q,\pi \right)\in P\left(\Theta \right)\times A^{r}.$$

Remark 2.4 For fixed q∈P(Θ), the problem

(8) $$\tilde{V}\left(q\right):=\underset{\alpha \in A}{\inf}V\left(q,\alpha \right)$$

is a dynamic Bayesian optimization with the prior q. Then Theorem 2.1 indicates that the exact optimal regret bound $R=inf_{\pi \in A^{r}}sup_{\theta \in \Theta }V\left(\theta ,\pi \right)$ for the MAB problem is given by the Bayesian optimisation under the worst prior $sup_{q\in P(\Theta )}\tilde{V}\left(q\right)$.

Example 2.2 Consider the example in which there are K coins. The probabilities to get a head (and thus receive $1) for each toss are $v_{1},\cdots ,v_{K}$ for these coins, but we do not know which probability $v_{i}$ corresponds to which coin. That is, Θ consists of all the permutations of (1,2,⋯,K), which is a finite set. In this case, (as can be expected) the uniform distribution on Θ would be an optimal solution for the problem $sup_{q\in P(\Theta )}\tilde{V}\left(q\right)$. Indeed, suppose $\hat{q}$ is an optimal solution for $sup_{q\in P(\Theta )}\tilde{V}\left(q\right)$. We may characterize $\hat{q}$ by the probability $r_{\theta }$ on each point θ∈Θ, and denote $\hat{q}∼{\left(r_{\theta }\right)}_{\theta \in \Theta }$. By symmetry, $\bar{q}∼{\left({\bar{r}}_{\theta }\right)}_{\theta \in \Theta }$ is also optimal for $sup_{q\in P(\Theta )}\tilde{V}\left(q\right)$ with ${\left({\bar{r}}_{\theta }\right)}_{\theta \in \Theta }$ being any permutation of ${\left(r_{\theta }\right)}_{\theta \in \Theta }$. Since the map $q\mapsto \tilde{V}\left(q\right)$ is concave, the average of these $\bar{q}$, which is the uniform distribution on Θ, would also be optimal for $sup_{q\in P(\Theta )}\tilde{V}\left(q\right)$.

Remark 2.5 The problem $\tilde{V}\left(q\right)$ in (8) can be solved directly by dynamic programming, and the values $\left\{\tilde{V}\left(q\right),\;q\in P\left(\Theta \right)\right\}$ are related by the dynamic programming equation (and thus in theory can be obtained simultaneously). For instance, in the case T=∞, $\tilde{V}$ solves the equation

(9) $$\tilde{V}\left(q\right)=e\left(q\right)+\underset{k\in K}{\inf}\int_{\Theta }\int_{R}\left(-z+\delta \tilde{V}\left(q{|}_{k,z}\right)\right)p_{k}^{\theta }\left(dz\right)q\left(d\theta \right),\;q\in P\left(\Theta \right),$$

where $e\left(q\right):=\int_{\Theta }E^{\theta }\left[X_{0}\left(a^{*}\left(\theta \right)\right)\right]q\left(d\theta \right)$, and ${q|}_{k,z}\in P\left(\Theta \right)$ is the posterior given (k,z), i.e.,

$${q|}_{k,z}\left(d\theta \right)=\frac{f_{k}^{\theta }\left(z\right)q\left(d\theta \right)}{\int_{\Theta }f_{k}^{\tilde{\theta }}\left(z\right)q\left(d\tilde{\theta }\right)}.$$

(It can be easily shown by the contraction mapping theorem that Eq. (9) has a unique solution.) The case for T<∞ is similar.

Remark 2.6 By Remark 2.2 and (7), it is possible that

$$\underset{\alpha \in A}{\inf}\underset{q\in P(\Theta )}{\sup}V\left(q,\alpha \right)>\underset{q\in P(\Theta )}{\sup}\underset{\alpha \in A}{\inf}V\left(q,\alpha \right).$$

Since $\sup_{\theta \in \Theta }\inf_{\pi \in A^{r}}V\left(\theta ,\pi \right)=0$, it is also possible that

$$\underset{\pi \in A^{r}}{\inf}\underset{\theta \in \Theta }{\sup}V\left(\theta ,\pi \right)>\underset{\theta \in \Theta }{\sup}\underset{\pi \in A^{r}}{\inf}V\left(\theta ,\pi \right).$$

This can be seen from the example in Remark 2.2 in which $R=\inf_{\pi \in A^{r}}\sup_{\theta \in \Theta }V\left(\theta ,\pi \right)=\frac{1}{6}$.

Remark 2.7 In general we do not have the uniqueness of a saddle point. This can be seen from the following example.

Example 2.3 Assume there are two arms. The outcome for one arm is always 1/2, and for the other arm is Bernoulli distributed with mean 1/2. We do not know which arm corresponds to which distribution. That is, we have Θ={1,2}. Consider the single-period case T=0. Then any $\left(q,\pi \right)\in P\left(\Theta \right)\times A^{r}$ is a saddle point.

Remark 2.8 Suppose we have obtained the optimizer $q^{*}\in P\left(\Theta \right)$ for the problem $sup_{q\in P(\Theta )}\tilde{V}\left(q\right)$ (and assume it is the same $q^{*}$ as in the saddle point). We further solve the problem $inf_{\pi \in A^{r}}V\left(q^{*},\pi \right)$ and get an optimizer $\hat{\pi }\in A^{r}$. There is no guarantee that $\hat{\pi }=\pi^{*}$ where $\pi^{*}$ is from the saddle point in Theorem 2.1, nor that $\hat{\pi }$ is an optimal solution for the original problem (6). Consider the two-coin one-period example from Remark 2.2. It can be easily shown that $q^{*}∼\left(1/2,1/2\right)$ is the unique optimizer for $sup_{q\in P(\Theta )}\tilde{V}\left(q\right)$, and $\hat{\pi }={\hat{\pi }}_{0}=\left(\pi_{0}\left(1\right),\pi_{0}\left(2\right)\right)=\left(1,0\right)$ is optimal for $inf_{\pi \in A^{r}}V\left(q^{*},\pi \right)$. However, $\hat{\pi }$ is obviously not optimal for $inf_{\pi \in A^{r}}sup_{\theta \in \Theta }V\left(\theta ,\pi \right)$.

To resolve this dilemma, in the next subsection we will modify the cost function a little bit, so that we could get a unique optimizer $q^{*}$ for $sup_{q\in P(\Theta )}{\tilde{V}}^{\eta ,\lambda }\left(q\right)$ and a unique optimizer $\pi^{*}$ for $inf_{\pi \in A^{r}}V^{\eta ,\lambda }\left(q^{*},\pi \right)$ and $inf_{\pi \in A^{r}}sup_{\theta \in \Theta }V^{\eta ,\lambda }\left(\theta ,\pi \right)$. Here ${\tilde{V}}^{\eta ,\lambda }$ and $V^{\eta ,\lambda }$ are the modifications of $\tilde{V}$ and V. Moreover, the obtained $\pi^{*}$ will be almost optimal for the original problem (6).

### 2.2 Second main result

Let ψ:P(Θ)↦[0,∞) be a bounded, continuous and strictly convex function. Such function ψ can be constructed as stated in the following result when $\Theta \subset R^{d}$ or $\Theta \subset R^{\infty }:=R\times R\times \cdots$.

Proposition 2.1 *For* d∈N*, define* $\psi_{d}:\;P\left(R^{d}\right)\mapsto \left[0,\infty \right)$*,*

$$\psi_{d}\left(q\right):=\int_{R^{d}}e^{-c_{d}∥z∥}F_{q}^{2}\left(z\right)dz,\;q\in P\left(R^{d}\right),$$

*where* ∥z∥ *is the Euclidean norm of* $z=\left(z_{1},\cdots ,z_{d}\right)\in R^{d}$*,* $F_{q}\left(z\right):=q\left(\Pi_{i=1}^{d}\left(-\infty ,z_{i}\right]\right)$ *is the cumulative distribution function of* q*, and* $c_{d}>0$ *is a constant such that* $\int_{R^{d}}e^{-c_{d}∥z∥}dz\leq 1$*. Let* $\psi_{\infty }:\;P\left(R^{\infty }\right)\mapsto \left[0,\infty \right)$*,*

$$\psi_{\infty }\left(q\right):=\sum_{d=1}^{\infty }\frac{1}{2^{d}}\psi_{d}\left(q_{d}\right),\;q\in P\left(R^{\infty }\right),$$

*where* $q_{d}$ *is the marginal distribution of* q *for the first* d *components. Then* $\psi_{d}$ *and* $\psi_{\infty }$ *are bounded, continuous and strictly convex.*

Recall Δ defined in (2). For $u=(u_{1},\cdots ,u_{k})\in \Delta$, define its Shannon’s entropy

$$\Gamma \left(u\right):=-\sum_{k\in K}u_{k}\ln\left(u_{k}\right).$$

For $\pi ={\left(\pi_{t}\right)}_{t\in N}$, denote

$$\Gamma \left(\pi_{t}\right)=\Gamma \left(\pi_{t}\left(H_{t}\right)\right)=-\left(\sum_{k\in K}\pi_{t}\left(k\right)\ln\left(\pi_{t}\left(k\right)\right)\right)\left(H_{t}\right).$$

For η,λ≥0 and $\left(q,\pi \right)\in P\left(\Theta \right)\times A^{r}$, denote

$$\begin{array}{cc} V^{\eta ,\lambda }\left(q,\pi \right) & :=V\left(q,\pi \right)-\lambda \int_{\Theta }E^{\theta ,\pi }\left[\sum_{t=0}^{T}\delta^{t}\Gamma \left(\pi_{t}\right)\right]q\left(d\theta \right)-\eta \psi \left(q\right)\\ & =\int_{\Theta }E^{\theta ,\pi }\left[\sum_{t=0}^{T}\delta^{t}\left(X_{t}\left(a^{*}\left(\theta \right)\right)-X_{t}\left(A_{t}\right)-\lambda \Gamma \left(\pi_{t}\right)\right)\right]q\left(d\theta \right)-\eta \psi \left(q\right). \end{array}$$

In addition to Assumption 2.1, we need the following assumption for our second main result.

Assumption 2.2

(i) For each k∈K, the support of $p_{k}^{*}$, denoted by $S_{k}:=\text{supp}\left(p^{*}\right)$, is compact.
(ii) There exists a constant $\underset{‾}{c}>0$ such that $f_{k}^{\theta }\geq \underset{‾}{c}$ on $S_{k}$ for any θ∈Θ and k∈K.
(iii) There exists a modulus of continuity function ρ such that

(10) $$|f_{k}^{\theta }\left(x\right)-f_{k}^{\theta }\left(y\right)|\leq \rho (|x-y|),\;\forall \;x,y\in S_{k},\;\theta \in \Theta ,\;k\in K.$$

Example 2.4 If X is finitely supported as in Example 2.1, then Assumption 2.2 (i)(iii) are satisfied.

Example 2.5 Let $\Theta =\Pi_{k=1}^{K}\left[l_{k},r_{k}\right]$ with $0<l_{k}\leq r_{k}<1$. For $\theta =(\theta_{1},\cdots ,\theta_{K})\in \Theta$, let $p_{k}^{\theta }$ be the distribution of $X_{0}\left(k\right)$ such that it is dominated by the Lebesgue measure L on [0,1], with the density function given by

$${\tilde{f}}_{k}^{\theta_{k}}=\frac{dp_{k}^{\theta }}{dL}=\left\{ \begin{array}{cc} \frac{2x}{\theta_{k}}, & x\in [0,\theta_{k}],\\ \frac{2(1-x)}{1-\theta_{k}}, & x\in (\theta_{k},1]. \end{array}\right.$$

If we choose the Lebesgue measure L as the reference measure, i.e., $p_{k}^{*}:=L$, then Assumption 2.2 (ii) will be violated. On the other hand, if we set $p_{k}^{*}:=p_{k}^{\theta^{*}}$ with $\theta^{*}\in \Theta$, then Assumption 2.2 (ii) is satisfied. We can also check that in this case all the conditions in Assumptions 2.1 and 2.2 are satisfied.

Remark 2.9 Assumption 2.2 (iii) can be weakened to the following condition: There exist finitely many disjoint intervals $\left(l_{k}^{i},r_{k}^{i}\right)\subset S_{k}$, $i=1,\cdots ,n_{k}$, such that $S_{k}=\cup_{i=1}^{n_{k}}\left[l_{k}^{i},r_{k}^{i}\right]$ and (10) holds on each $\left(l_{k}^{i},r_{k}^{i}\right)$. With this weakened assumption, Theorem 2.2 still holds and the proof still goes through (with some trivial adjustment).

Example 2.6 Let $\Theta =\Theta_{1}\times \cdots \times \Theta_{K}$ with $\Theta_{k}=\left\{\left(m,\sigma^{2}\right)\in R^{2}:{\underset{‾}{m}}_{k}\leq m\leq {\bar{m}}_{k},\;{\underset{‾}{\sigma }}_{k}^{2}\leq \sigma^{2}\leq {\bar{\sigma }}_{k}^{2}\right\}$. Here ${\underset{‾}{m}}_{k},{\bar{m}}_{k},{\underset{‾}{\sigma }}^{2}>0,{\bar{\sigma }}^{2}>0$ are given constants. Let ${\tilde{p}}_{k}^{m,\sigma^{2}}\in P\left(R\right)$ be the probability measure induced by the normal distribution with mean m and variance $\sigma^{2}$. For each $\theta =\left(\theta_{1},\cdots ,\theta_{k}\right)=\left(\left(m_{1},\sigma_{1}^{2}\right)\cdots ,\left(m_{K},\sigma_{K}^{2}\right)\right)\in \Theta$, let $p_{k}^{\theta }=p_{k}^{m_{k},\sigma_{k}^{2}}\in P\left(\left[0,1\right]\right)$, where

$$p_{k}^{m,\sigma^{2}}\left(\left(-\infty ,x\right]\right):=\left\{ \begin{array}{cc} 0, & x<0,\\ {\tilde{p}}_{k}^{m,\sigma^{2}}\left(\left(-\infty ,x\right]\right), & 0\leq x<1,\\ 1, & x\geq 1. \end{array}\right.$$

Let $p_{k}^{*}:=p_{k}^{0,1}$. Then Assumption 2.2 (iii) does not hold, but the weakened condition in Remark 2.9 is satisfied. We can also show that all the other conditions in Assumptions 2.1 and 2.2 are satisfied.

Below is our second main result.

Theorem 2.2 *Let* Assumptions 2.1 *and* 2.2 *hold, and* η≥0,λ>0*. Then there exists a saddle point* $\left(q^{*},\pi^{*}\right)\in P\left(\Theta \right)\times A^{r}$ *for* $V^{\eta ,\lambda }$*, i.e.,*

(11) $$V^{\eta ,\lambda }\left(q,\pi^{*}\right)\leq V^{\eta ,\lambda }\left(q^{*},\pi^{*}\right)\leq V^{\eta ,\lambda }\left(q^{*},\pi \right),\;\forall \;\left(q,\pi \right)\in P\left(\Theta \right)\times A^{r}.$$

*Moreover,* $\pi^{*}$ *is the unique optimizer for the problem* $inf_{\pi \in A^{r}}V^{\eta ,\lambda }\left(q^{*},\pi \right)$*. If in addition* η>0*, then the saddle point is unique.*

Denote

$$C_{1}:=sup_{q\in P(\Theta )}\psi \left(q\right)\in \left(0,\infty \right)\;\text{and}\;C_{2}:=\max_{u\in \Delta }\Gamma \left(u\right)\cdot \sum_{t=0}^{T}\delta^{t}\in \left(0,\infty \right).$$

We have the following result.^1^

Corollary 2.1 *For* η≥0 *and* λ>0*, let* $\left(q^{*},\pi^{*}\right)$ *be the saddle point for* $V^{\eta ,\lambda }$ *from* Theorem 2.2*. Then* $\left(q^{*},\pi^{*}\right)$ *is a* $\left(C_{1}\eta +C_{2}\lambda \right)$*-saddle point for* V*. That is,*

$$\begin{array}{ccc} & & V\left(q,\pi^{*}\right)-\left(C_{1}\eta +C_{2}\lambda \right)\leq V\left(q^{*},\pi^{*}\right)\leq V\left(q^{*},\pi \right)+\left(C_{1}\eta +C_{2}\lambda \right),\\ & & \forall \;\left(q,\pi \right)\in P\left(\Theta \right)\times A^{r}. \end{array}$$

*In particular,* $\pi^{*}$ *is* $2(C_{1}\eta +C_{2}\lambda )$*-optimal for* $inf_{\pi \in A^{r}}sup_{q\in P(\Theta )}V\left(q,\pi \right)$*, i.e.,*

$$\underset{q\in P(\Theta )}{\sup}V\left(q,\pi^{*}\right)\leq \underset{\pi \in A^{r}}{\inf}\underset{q\in P(\Theta )}{\sup}V\left(q,\pi \right)+2\left(C_{1}\eta +C_{2}\lambda \right).$$

Remark 2.10 Similar to Remark 2.5, for q∈P(Θ) the problem

$${\tilde{V}}^{\eta ,\lambda }\left(q\right):=inf_{\pi \in A^{r}}V^{\eta ,\lambda }\left(q,\pi \right)$$

can be solved by the dynamic programming principle, and the values $\left\{{\tilde{V}}^{\eta ,\lambda }\left(q\right),\;q\in P\left(\Theta \right)\right\}$ are related by the dynamic programming equation. For η>0, since the map $q\mapsto {\tilde{V}}^{\eta ,\lambda }\left(q\right)$ is strictly concave, the maximizer is unique and thus must coincide with $q^{*}$ from Theorem 2.2. Now suppose we have already obtained $q^{*}$. Theorem 2.2 indicates that the unique solution to the problem $inf_{\pi \in A^{r}}V^{\eta ,\lambda }\left(q^{*},\pi \right)$ is also optimal for the problem $inf_{\pi \in A^{r}}sup_{q\in P(\Theta )}V^{\eta ,\lambda }\left(q,\pi \right)$. This resolves the dilemma mentioned in Remark 2.8.

Note that in theory, the saddle point $\left(q^{*},\pi^{*}\right)$ from Theorem 2.2 can be solved simultaneously from the dynamic programming equation for $\left\{{\tilde{V}}^{\eta ,\lambda }\left(q\right),\;q\in P\left(\Theta \right)\right\}$, since the optimal control can also be obtained from the equation. In addition, by Corollary 2.1 the solution $\left(q^{*},\pi^{*}\right)$, in particular $\pi^{*}$, is almost optimal for the original problem $inf_{\pi \in A^{r}}sup_{q\in P(\Theta )}V\left(q,\pi \right)$ by choosing η,λ>0 small enough.

Remark 2.11 When η=0, we may not have the uniqueness of a saddle point. For instance, in Example 2.3 with λ>0, we can see that for $\pi_{0}^{*}=\left(1/2,1/2\right)$ and any q∈P(Θ), $\left(q,\pi^{*}\right)$ is a saddle point for $V^{0,\lambda }$.

For $\pi ,\pi^{\prime }\in A^{r}$, define the distance

(12) $$d_{A^{r}}\left(\pi ,\pi^{\prime }\right):=\sum_{t=0}^{T}\frac{1}{2^{t}}\underset{k\in K,h_{t-1}\in H_{t-1}}{\sup}\left|\pi_{t}\left(k\right)\left(h_{t-1}\right)-\pi_{t}^{\prime }\left(k\right)\left(h_{t-1}\right)\right|.$$

We use the topology on $A^{r}$ induced by $d_{A^{r}}$. Letting η,λ→0, we have the following result.

Proposition 2.2 *For* $\eta_{n}\geq 0$ *and* $\lambda_{n}>0$*, let* $\left(q^{n},\pi^{n}\right)\in P\left(\Theta \right)\times A^{r}$ *be a saddle point for* $V^{\eta_{n},\lambda_{n}}$*. Suppose* $\eta_{n},\lambda_{n}\to 0$ *and* $\left(q^{n},\pi^{n}\right)\to \left(\hat{q},\hat{\pi }\right)$ *as* n→∞*. Then* $\left(\hat{q},\hat{\pi }\right)$ *is a saddle point for* V*.*

Remark 2.12 The proof of Theorem 2.2, Corollary 2.1 and Proposition 2.2 provides an alternative method to show Theorem 2.1 (under more restrictive assumptions).

## 3 Proofs for results in Section 2

In this section, we provide the proofs for the results in Section 2. We present the argument for the infinite-time horizon case T=∞. The proof for the finite-horizon case T<∞ is similar and in fact easier, and thus will be omitted. That is, we assume T=∞ throughout this section.

The overall idea to prove Theorem 2.1 is to apply the minimax theorem ([15, Corollary 2]) to show (7) and Kakutani-Fan’s fixed-point theorem ([16, Theorem 1]) to show the existence of a saddle point. A technical difficulty to apply the minimax (and Kakutani-Fan’s fixed-point) theorem is that, the map π↦V(q,π) is not convex for $\pi \in A^{r}$ (see Remark 3.1 and Example 3.1). To resolve this dilemma, instead of $\pi \in A^{r}$ we work on μ∈Ξ (defined in (13)), the law of ${\left(A_{t}\right)}_{t}$ conditioned on the path of ${\left(X_{t}\right)}_{t}$, as the map μ↦V(q,μ) is linear. We will show in Lemma 3.1 that there is a one-to-one correspondence between μ∈Ξ and $\pi \in A^{r}$ in certain sense. Therefore, it indeed suffices for us to focus on μ∈Ξ. In Lemma 3.3 we show that the map (q,μ)↦V(q,μ) is continuous for (q,μ)∈P(Θ)×Ξ, which is a key step to apply the minimax and Kakutani-Fan’s fixed-point theorems.

For the rest of the paper, we often use the notation $a_{t}:=\left(a_{0},\cdots ,a_{t}\right)$ for $a_{0},\cdots ,a_{t}\in K$, $x_{t}:=\left(x_{0},\cdots ,x_{t}\right)$ for $x_{0},\cdots ,x_{t}\in R^{K}$, $X_{t}:=\left(X_{0},\cdots ,X_{t}\right)$, $A_{t}:=\left(A_{0},\cdots ,A_{t}\right)$,

$$x_{t}\left(a_{t}\right):=\left(x_{0}\left(a_{0}\right),\cdots ,x_{t}\left(a_{t}\right)\right),\;\text{and}\;X_{t}\left(a_{t}\right):=\left(X_{0}\left(a_{0}\right),\cdots ,X_{t}\left(a_{t}\right)\right).$$

Let Ξ be a set of transition kernels from $\left(\Omega^{X},B\left(\Omega^{X}\right)\right)$ to $\left(\Omega^{A},B\left(\Omega^{A}\right)\right)$ defined by

(13) $$\begin{array}{cc} \Xi := & \{\mu_{\cdot }:\Omega^{X}\mapsto P\left(\Omega^{A}\right)\;\text{Borel}\;\text{measurable}:\\ & \mu_{x}\left(A_{t}=a_{t}\right)=\mu_{x^{\prime }}\left(A_{t}=a_{t}\right)\;\text{if}\;x_{t-1}\left(a_{t-1}\right)\\ & =x_{t-1}^{\prime }\left(a_{t-1}\right),\;\forall \;x,x^{\prime }\in \Omega^{X},\;a_{t}\in K^{t+1},\;\;t\in N\}. \end{array}$$

For μ∈Ξ, with a little abuse of notation we denote $P^{\theta ,\mu }:=P^{\theta }⊗\mu$.

Lemma 3.1 *For any* $\pi \in A^{r}$*, We have* $P^{\pi }\in \Xi$*. Conversely, for any* μ∈Ξ *there exists* $\pi \in A^{r}$ *such that* $P^{\pi }=\mu$*.*

Proof Let $\pi \in A^{r}$. For $x\in \Omega^{X}$ and $a_{t}=\left(a_{0},\cdots ,a_{t}\right)\in K^{t+1}$,

$$\begin{array}{cc} P_{x}^{\pi }\left(A_{t}=a_{t}\right) & =P_{x}^{\pi }\left(A_{0}=a_{0}\right)\cdots P_{x}^{\pi }\left(A_{t}=a_{t}\;|\;A_{t-1}=a_{t-1}\right)\\ & =\pi_{0}\cdot \pi_{1}\left(a_{0},x_{0}\left(a_{0}\right)\right)\cdots \pi_{t}\left(a_{t-1},x_{t-1}\left(a_{t-1}\right)\right). \end{array}$$

We can see that $P_{x}^{\pi }\left(A_{t}=a_{t}\right)$ depend on x only through $x_{t-1}\left(a_{t-1}\right)$. Hence $P^{\pi }\in \Xi$. Conversely, take μ∈Ξ. For $x\in \Omega^{X}$ and $a_{t}\in K^{t+1}$, we may define π by

(14) $$\pi_{t}\left(a_{t}\right)\left(x_{t-1}\left(a_{t-1}\right)\right)=\mu_{x}\left(A_{t}=a_{t}\;|\;A_{t-1}=a_{t-1}\right)=\frac{\mu_{x}\left(A_{t}=a_{t}\right)}{\mu_{x}\left(A_{t-1}=a_{t-1}\right)},$$

where we set $\frac{0}{0}:=\frac{1}{K}$. Note the right side of (14) depends on x only through $x_{t-1}\left(a_{t-1}\right)$ by the definition of Ξ, and thus $\pi \in A^{r}$ is well defined. Obviously, we have $P^{\pi }=\mu$. □

With a bit of abuse of notation, for μ∈Ξ denote

$$V\left(q,\mu \right):=\int_{\Theta }E^{\theta ,\mu }\left[\sum_{t=0}^{T}\delta^{t}\left(X_{t}\left(a^{*}\left(\theta \right)\right)-X_{t}\left(A_{t}\right)\right)\right]q\left(d\theta \right).$$

By Lemma 3.1, we have that

$$\begin{array}{ccc} & & \underset{\pi \in A^{r}}{\inf}\underset{q\in P(\Theta )}{\sup}V\left(q,\pi \right)=\underset{\mu \in \Xi }{\inf}\underset{q\in P(\Theta )}{\sup}V\left(q,\mu \right)\\ & & \text{and}\;\underset{q\in P(\Theta )}{\sup}\underset{\pi \in A^{r}}{\inf}V\left(q,\pi \right)=\underset{q\in P(\Theta )}{\sup}inf_{\mu \in \Xi }V\left(q,\mu \right). \end{array}$$

Remark 3.1 We will work on μ∈Ξ instead of $\pi \in A^{r}$ to show Theorem 2.1, by using the minimax and Kakutani-Fan’s fixed point theorem. The reason is that the map π↦V(θ,π) is not linear and even not convex, as shown by the following example.

Example 3.1 Suppose there are two arms, i.e., K=2. Let Θ={θ} be a singleton, under which arm 1 always gives outcome 1, and arm 2 always gives outcome 0. Let T=1 and δ=1. Define $\pi ,\pi^{\prime }\in A^{r}$,

$$\;\pi_{0}=\left(1,0\right),\;\pi_{1}\equiv \left(1,0\right),\;\text{and}\;\pi_{0}^{\prime }=\left(0,1\right),\;\pi_{1}^{\prime }\left(h_{0}\right)=\left\{ \begin{array}{cc} (1,0), & h_{0}=\left(1,1\right),\\ (0,1), & h_{0}=\left(2,0\right). \end{array}\right.$$

Then for $\tilde{\pi }:=\frac{1}{2}\left(\pi +\pi^{\prime }\right)$,

$${\tilde{\pi }}_{0}=\left(1/2,1/2\right),\;{\tilde{\pi }}_{1}\left(h_{0}\right)=\left\{ \begin{array}{cc} (1,0), & h_{0}=\left(1,1\right),\\ (1/2,1/2), & h_{0}=\left(2,0\right). \end{array}\right.$$

We can compute that

$$\frac{1}{2}V\left(\theta ,\pi \right)+\frac{1}{2}V\left(\theta ,\pi^{\prime }\right)=1<\frac{5}{4}=V\left(\theta ,\frac{1}{2}\left(\pi +\pi^{\prime }\right)\right).$$

This implies the map π↦V(θ,π) is not convex.

Let $p^{*}:=p_{1}^{*}\times \cdots \times p_{K}^{*}\in P\left(R^{K}\right)$ and $P^{*}:=p^{*}\times p^{*}\times \cdots \in P\left(\Omega_{X}\right)$. Denote $E^{*}\left[\cdot \right]$ the expectation under the probability measure $P^{*}$. We have the following result.

Lemma 3.2 *Let* Assumption 2.1 *hold. Then for any fixed* μ∈Ξ*, the map* θ↦V(θ,μ) *is continuous for* θ∈Θ*.*

Proof Fix μ∈Ξ and take $\theta_{n},\theta \in \Theta$ with $\theta_{n}\to \theta$. Let ε>0. Thanks to Assumption 2.1, we have that

$$\begin{array}{ccc} & & E^{\theta ,\mu }\left[\right|X_{t}\left(a^{*}\left(\theta_{n}\right)\right)\left|+\right|X_{t}\left(A_{t}\right)\left|\right]\leq 2E^{\theta }\left[\sum_{k\in K}\left|X_{0}\left(k\right)\right|\right]\\ & & =2E^{*}\left[\sum_{k\in K}f_{k}\left(X_{0}\left(K\right)\right)\left|X_{0}\left(k\right)\right|\right]\leq 2K{\tilde{C}}^{2}<\infty . \end{array}$$

Let $T_{0}\in N$ such that

(15) $$\sum_{t=T_{0}+1}^{\infty }\delta^{t}2K{\tilde{C}}^{2}<\varepsilon .$$

Note for each t∈N,

$$\begin{array}{cc} E^{\theta_{n}}\left[X_{t}\left(a^{*}\left(\theta_{n}\right)\right)\right] & =\max_{k\in K}\left\{E^{\theta_{n}}\left[X_{t}\left(1\right)\right],\cdots ,E^{\theta_{n}}\left[X_{t}\left(k\right)\right]\right\}\\ & =\max_{k\in K}\left\{E^{*}\left[f_{1}^{\theta_{n}}\left(X_{t}\left(1\right)\right)X_{t}\left(1\right)\right],\cdots ,E^{*}\left[f_{k}^{\theta_{n}}\left(X_{t}\left(k\right)\right)X_{t}\left(k\right)\right]\right\}\\ & \to \max_{k\in K}\left\{E^{*}\left[f_{1}^{\theta }\left(X_{t}\left(1\right)\right)X_{t}\left(1\right)\right],\cdots ,E^{*}\left[f_{k}^{\theta }\left(X_{t}\left(k\right)\right)X_{t}\left(k\right)\right]\right\}\\ & =E^{\theta }\left[X_{t}\left(a^{*}\left(\theta_{n}\right)\right)\right], \end{array}$$

where the convergence follows from the dominated convergence theorem thanks to Assumption 2.1. We have that

(16) $$\begin{array}{ccc} E^{\theta_{n},\mu }\left[X_{t}\left(A_{t}\right)\right] & = & \sum_{k=1}^{K}E^{\theta_{n},\mu }\left[X_{t}\left(k\right)1_{\left\{A_{t}=k\right\}}\right]\\ & = & \sum_{k=1}^{K}E^{\theta_{n},\mu }\left[E^{\theta_{n},\mu }\left[X_{t}\left(k\right)1_{\left\{A_{t}=k\right\}}\;|\;X\right]\right]\\ & = & \sum_{k=1}^{K}E^{\theta_{n}}\left[X_{t}\left(k\right)\mu_{X}\left(A_{t}=k\right)\right]\\ & = & \sum_{k=1}^{K}E^{\theta_{n}}\left[X_{t}\left(k\right)\right]\cdot E^{\theta_{n}}\left[\mu_{X}\left(A_{t}=k\right)\right], \end{array}$$

where the last equality follows from the fact that $\mu_{X}\left(A_{t}=k\right)$ depend on X only through $X_{t-1}$ and thus is independent of $X_{t}$. We have that

(17) $$\begin{array}{cc} & E^{\theta_{n}}\left[\mu_{X}\left(A_{t}=k\right)\right]=\sum_{a_{t-1}\in K^{t}}E^{\theta_{n}}\left[\mu_{X}\left(A_{t-1}=a_{t-1},A_{t}=k\right)\right]\\ & \;=\sum_{a_{t-1}\in K^{t}}E^{*}\left[f_{0}^{\theta_{n}}\left(X_{0}\left(a_{0}\right)\right)\cdots f_{t-1}^{\theta^{n}}\left(X_{t-1}\left(a_{t-1}\right)\right)\mu_{X}\left(A_{t-1}=a_{t-1},A_{t}=k\right)\right]\\ & \;\to \sum_{a_{t-1}\in K^{t}}E^{*}\left[f_{0}^{\theta }\left(X_{0}\left(a_{0}\right)\right)\cdots f_{t-1}^{\theta }\left(X_{t-1}\left(a_{t-1}\right)\right)\mu_{X}\left(A_{t-1}=a_{t-1},A_{t}=k\right)\right]\\ & \;=E^{\theta }\left[\mu_{X}\left(A_{t}=k\right)\right], \end{array}$$

where for (17) we use the fact that $\mu_{X}\left(A_{t-1}=a_{t-1},A_{t}=k\right)$ depends on X only through $X_{t-1}\left(a_{t-1}\right)$, and the convergence follows from the dominated convergence theorem. As a result, we have that

$$E^{\theta_{n},\mu }\left[X_{t}\left(A_{t}\right)\right]\to E^{\theta ,\mu }\left[X_{t}\left(A_{t}\right)\right],\;t\in N.$$

Then we may choose N∈N such that for any n>N, it holds that

$$\left|E^{\theta_{n},\mu }\left[\sum_{t=0}^{T_{0}}\delta^{t}\left(X_{t}\left(a^{*}\left(\theta_{n}\right)\right)-X_{t}\left(A_{t}\right)\right)\right]-E^{\theta ,\mu }\left[\sum_{t=0}^{T_{0}}\delta^{t}\left(X_{t}\left(a^{*}\left(\theta \right)\right)-X_{t}\left(A_{t}\right)\right)\right]\right|<\varepsilon .$$

Then for any n>N,

$$\left|E^{\theta_{n},\mu }\left[\sum_{t=0}^{\infty }\delta^{t}\left(X_{t}\left(a^{*}\left(\theta_{n}\right)\right)-X_{t}\left(A_{t}\right)\right)\right]-E^{\theta ,\mu }\left[\sum_{t=0}^{\infty }\delta^{t}\left(X_{t}\left(a^{*}\left(\theta \right)\right)-X_{t}\left(A_{t}\right)\right)\right]\right|<3\varepsilon ,$$

and thus θ↦V(θ,μ) is continuous. □

For $\mu ,\mu^{\prime }\in \Xi$, define the distance

$$d_{\Xi }\left(\mu ,\mu^{\prime }\right):=\sum_{t=0}^{T}\frac{1}{2^{t}}sup_{x\in \Omega^{X},a_{t}\in K^{t+1}}\left|\mu_{x}\left(A_{t}=a_{t}\right)-\mu_{x}^{\prime }\left(A_{t}=a_{t}\right)\right|.$$

We use the topology on Ξ induced by $d_{\Xi }$.

Lemma 3.3 *Let* Assumption 2.1 *hold. For* (q,μ)∈P(Θ)×Ξ*, the map* (q,μ)↦V(q,μ) *is continuous.*

Proof Let $\left(q^{n},\mu^{n}\right),\left(q,\mu \right)\in P\left(\Theta \right)\times \Xi$ with $\left(q^{n},\mu^{n}\right)\to \left(q,\mu \right)$ as n→∞. We have

$$|V\left(q^{n},\mu^{n}\right)-V\left(q,\mu \right)|\leq |V\left(q^{n},\mu^{n}\right)-V\left(q^{n},\mu \right)|+|V\left(q^{n},\mu \right)-V\left(q,\mu \right)|.$$

By Lemma 3.2,

$$|V\left(q^{n},\mu \right)-V\left(q,\mu \right)|=\left|\int_{\Theta }V\left(\theta ,\mu \right)q^{n}\left(d\theta \right)-\int_{\Theta }V\left(\theta ,\mu \right)q\left(d\theta \right)\right|\to 0,\;n\to \infty .$$

Let us consider $|V\left(q^{n},\mu^{n}\right)-V\left(q^{n},\mu \right)|$. Let ε>0. Choose $T_{0}\in N$ such that (15) holds. For each θ∈Θ and t∈N, by (16) we have that

$$\begin{array}{cc} & \left|E^{\theta ,\mu^{n}}\left[X_{t}\left(A_{t}\right)\right]-E^{\theta ,\mu }\left[X_{t}\left(A_{t}\right)\right]\right|\\ & =\left|\sum_{k=1}^{K}E^{\theta }\left[X_{t}\left(k\right)\right]\cdot E^{\theta }\left[\mu_{X}^{n}\left(A_{t}=k\right)\right]-\sum_{k=1}^{K}E^{\theta }\left[X_{t}\left(k\right)\right]\cdot E^{\theta }\left[\mu_{X}\left(A_{t}=k\right)\right]\right|,\\ & \leq \sum_{k=1}^{K}E^{\theta }\left|X_{t}\left(k\right)\right|\cdot E^{\theta }\left[\left|\mu_{X}^{n}\left(A_{t}=k\right)-\mu_{X}\left(A_{t}=k\right)\right|\right]\\ & \leq K{\tilde{C}}^{2}{\left(2K\right)}^{t}d_{\Xi }\left(\mu^{n},\mu \right)\to 0,\;n\to \infty . \end{array}$$

Hence, we can choose N∈N independent of θ such that for any n>N,

$$\begin{array}{ccc} & & \left|E^{\theta ,\mu^{n}}\left[\sum_{t=0}^{T_{0}}\delta^{t}(X_{t}\left(a^{*}\left(\theta_{n}\right)-X_{t}\left(A_{t}\right)\right)\right]-E^{\theta ,\mu }\left[\sum_{t=0}^{T_{0}}\delta^{t}(X_{t}\left(a^{*}\left(\theta_{n}\right)-X_{t}\left(A_{t}\right)\right)\right]\right|\\ & & \;\;\;\;<\varepsilon ,\;\forall \;\theta \in \Theta . \end{array}$$

Then for any n>N,

$$|V\left(\theta ,\mu^{n}\right)-V\left(\theta ,\mu \right)|<3\varepsilon ,$$

and thus

$$\begin{array}{cc} |V\left(q^{n},\mu^{n}\right)-V\left(q^{n},\mu \right)| & =\left|\int_{\Theta }V\left(\theta ,\mu^{n}\right)q^{n}\left(d\theta \right)-\int_{\Theta }V\left(\theta ,\mu \right)q^{n}\left(d\theta \right)\right|\\ & \leq \int_{\Theta }\left|V\left(\theta ,\mu^{n}\right)-V\left(\theta ,\mu \right)\right|q^{n}\left(d\theta \right)\leq 3\varepsilon . \end{array}$$

As a result, we have $|V\left(q^{n},\mu^{n}\right)-V\left(q^{n},\mu \right)|\to 0$ and thus $V\left(q^{n},\mu^{n}\right)\to V\left(q,\mu \right)$ as n→∞. □

We are ready to provide the proof of Theorem 2.1.

Proof of Theorem 2.1 It is easy to show the first equality in (7). The third equality is also obvious since for any fixed q∈P(Θ), the optimal solution for $inf_{\pi \in A^{r}}V\left(q,\pi \right)$ is attained at a pure strategy as can be seen from the dynamic programming equation.

Assume Θ is compact and thus P(Θ) is compact. For q∈P(Θ) and μ∈Ξ, the maps q↦V(q,μ) and μ↦V(q,μ) are linear and continuous. Then we can apply the minimax theorem (e.g., [15, Corollary 2]) and get

(18) $$\underset{\mu \in \Xi }{\inf}\underset{q\in P(\Theta )}{\sup}V\left(q,\mu \right)=\underset{q\in P(\Theta )}{\sup}\underset{\mu \in \Xi }{\inf}V\left(q,\mu \right).$$

Thanks to Lemma 3.1, we have

$$\begin{array}{ccc} & & \underset{\pi \in A^{r}}{\inf}\underset{q\in P(\Theta )}{\sup}V\left(q,\pi \right)=\underset{\mu \in \Xi }{\inf}\underset{q\in P(\Theta )}{\sup}V\left(q,\mu \right)\\ & & \;=\underset{q\in P(\Theta )}{\sup}\underset{\mu \in \Xi }{\inf}V\left(q,\mu \right)=\underset{q\in P(\Theta )}{\sup}\underset{\pi \in A^{r}}{\inf}V\left(q,\pi \right). \end{array}$$

If X is finitely supported, then w.l.o.g. we may only focus on the path space $\left(\Pi_{k\in K}S_{k}\right)\times \left(\Pi_{k\in K}S_{k}\right)\times \cdots$ for X. In that case, Ξ is compact under $d_{\Xi }$. This can be seen from the fact that for each μ∈Ξ, $\mu_{x}\left(A_{t}=a_{t}\right)$ depends on x only through $x_{t-1}$ and there are only finitely many possible paths for $x_{t-1}$. Thanks to the compactness of Ξ, we can again apply the minimax theorem and get (18) and thus (7).

Now we assume both Θ is compact and X is finitely supported. Define the set-valued map $\Phi :P\left(\Theta \right)\times \Xi \mapsto 2^{P(\Theta )\times \Xi }$,

$$\Phi \left(q,\mu \right):=\left\{\left(\tilde{q},\tilde{\mu }\right)\in P\left(\Theta \right)\times \Xi :\;\tilde{q}\in \text{arg}\max_{\hat{q}\in P\left(\Theta \right)}V\left(\hat{q},\mu \right),\;\tilde{\mu }\in arg\;min_{\hat{\mu }\in \Xi }V\left(q,\hat{\mu }\right)\right\}.$$

By Lemma 3.3, the set Φ(q,μ) is nonempty, compact and convex for any (q,μ)∈P(Θ)×Ξ. Take $\left(q^{n},\mu^{n}\right),\left(q,\mu \right),\left({\tilde{q}}^{n},{\tilde{\mu }}^{n}\right),\left(\tilde{q},\tilde{\mu }\right)\in P\left(\Theta \right)\times \Xi$ with $\left({\tilde{q}}^{n},{\tilde{\mu }}^{n}\right)\in \Phi \left(q^{n},\mu^{n}\right)$, $\left(q^{n},\mu^{n}\right)\to \left(q,\mu \right)$ and $\left({\tilde{q}}^{n},{\tilde{\mu }}^{n}\right)\to \left(\tilde{q},\tilde{\mu }\right)$. By the definition of Φ,

$$V\left({\tilde{q}}^{n},\mu^{n}\right)\geq V\left(\hat{q},\mu^{n}\right)\;\text{and}\;V\left(q^{n},{\tilde{\mu }}^{n}\right)\leq V\left(q^{n},\hat{\mu }\right),\;\forall \;\left(\hat{q},\hat{\mu }\right)\in P\left(\Theta \right)\times \Xi .$$

Letting n→∞, by Lemma 3.3 we have that

$$V\left(\tilde{q},\mu \right)\geq V\left(\hat{q},\mu \right)\;\text{and}\;V\left(q,\tilde{\mu }\right)\leq V\left(q,\hat{\mu }\right),\;\forall \;\left(\hat{q},\hat{\mu }\right)\in P\left(\Theta \right)\times \Xi .$$

Therefore, $\left(\tilde{q},\tilde{\mu }\right)\in \Phi \left(\mu ,q\right)$ and thus Φ(·) is upper-semicontinuous. By Kakutani-Fan’s fixed-point theorem (see e.g. [16, Theorem 1]), there exists $\left(q^{*},\mu^{*}\right)\in P\left(\Theta \right)\times \Xi$ such that

$$V\left(q,\mu^{*}\right)\leq V\left(q^{*},\mu^{*}\right)\leq V\left(q^{*},\mu \right),\;\forall \;\left(q,\mu \right)\in P\left(\Theta \right)\times \Xi .$$

Thanks to Lemma 3.1, we can find $\pi^{*}\in A^{r}$, such that

$$V\left(q,\pi^{*}\right)\leq V\left(q^{*},\pi^{*}\right)\leq V\left(q^{*},\pi \right),\;\forall \;\left(q,\pi \right)\in P\left(\Theta \right)\times A^{r}.$$

This completes the proof. □

Proof of Proposition 2.1 Consider $\psi_{d}$. Take $q,\tilde{q}\in P\left(R^{d}\right)$ and λ∈(0,1). We have that

$$\begin{array}{cc} \psi_{d}\left(\lambda q+\left(1-\lambda \right)\tilde{q}\right) & =\int_{R^{d}}e^{-c_{d}∥z∥}{\left(\lambda F_{q}\left(z\right)+\left(1-\lambda \right)F_{\tilde{q}}\left(z\right)\right)}^{2}dz\\ & \leq \int_{R^{d}}e^{-c_{d}∥z∥}\left(\lambda F_{q}^{2}\left(z\right)+\left(1-\lambda \right)F_{\tilde{q}}^{2}\left(z\right)\right)dz\\ & =\lambda \psi_{d}\left(q\right)+\left(1-\lambda \right)\psi_{d}\left(\tilde{q}\right). \end{array}$$

Note in the above the equality holds if and only if

$$\begin{array}{cc} & F_{q}\left(z\right)=F_{\tilde{q}}\left(z\right),\;\text{for}\;\text{a.e.}\;z\in R^{d}\;\text{(w.r.t.}\;\text{Lebesgue}\;\text{measure)}\\ & ⟺\;F_{q}\left(z\right)=F_{\tilde{q}}\left(z\right),\;\forall \;z\in R^{d}\;⟺\;q=\tilde{q}. \end{array}$$

Hence, ψ is strictly convex.

Let $q^{n},q\in P\left(R^{d}\right)$ with $q^{n}\to q$. Then $F_{q^{n}}\left(z\right)\to F_{q}\left(z\right)$ for a.e. $z\in R^{d}$. By the dominated convergence theorem,

$$\int_{R^{d}}e^{-c_{d}∥z∥}F_{q^{n}}^{2}\left(z\right)dz\to \int_{R^{d}}e^{-c_{d}∥z∥}F_{q}^{2}\left(z\right)dz,\;n\to \infty ,$$

which implies $\psi_{d}$ is continuous.

Now consider $\psi_{\infty }$. For $q,\tilde{q}\in P\left(R^{\infty }\right)$ and λ∈(0,1),

$$\begin{array}{cc} \psi_{\infty }\left(\lambda q+\left(1-\lambda \right)\tilde{q}\right) & =\sum_{d=1}^{\infty }\frac{1}{2^{d}}\psi_{d}\left(\lambda q_{d}+\left(1-\lambda \right){\tilde{q}}_{d}\right)\\ & \leq \sum_{d=1}^{\infty }\frac{1}{2^{d}}\left[\lambda \psi_{d}\left(q_{d}\right)+\left(1-\lambda \right)\psi_{d}\left({\tilde{q}}_{d}\right)\right]\\ & =\lambda \psi_{\infty }\left(q\right)+\left(1-\lambda \right)\psi_{\infty }\left(\tilde{q}\right). \end{array}$$

In the above the equality holds if and only if

$$\begin{array}{c} q_{d}={\tilde{q}}_{d},\;\forall \;d\in N\;⟺\;q=\tilde{q}. \end{array}$$

Consequently, ψ is strictly convex.

Take $q^{n},q\in P\left(R^{\infty }\right)$ with $q^{n}\to q$. We have $q_{d}^{n}\to q_{d}$ for any d∈N. Let ε>0. Choose D∈N such that $\sum_{d=D+1}^{\infty }\frac{1}{2^{d}}<\varepsilon$. Then choose N∈N such that for any n>N,

$$\left|\sum_{d=1}^{D}\frac{1}{2^{d}}\psi_{d}\left(p_{d}^{n}\right)-\sum_{d=1}^{D}\frac{1}{2^{d}}\psi_{d}\left(p_{d}\right)\right|<\varepsilon \;\Rightarrow \;\left|\psi_{\infty }\left(q^{n}\right)-\psi_{\infty }\left(q\right)\right|<3\varepsilon .$$

As a result, $\psi_{\infty }$ is continuous. □

We will use Kakutani-Fan’s fixed-point theorem to prove Theorem 2.2, by considering a set-valued mapping (q,π)↦Φ(q,π) (defined in (30)). The application of the fixed-point theorem requires the compactness of the underlying set for (q,π). However, the original set $A^{r}$ for π is not compact. To deal with this issue, we show in Lemma 3.5 that the minimizer for $inf_{\pi \in A^{r}}V^{\eta ,\lambda }\left(q,\pi \right)$ (equivalently, the maximizer for F(q) in (22)) is equicontinuous in certain sense. This enables us to apply the Arzela-Ascoli theorem and focus on a compact subset ${\bar{A}}^{r}\subset A^{r}$ (defined in (29)) for the set-valued mapping Φ. Another important step is to show the continuity of $\left(q,\pi \right)\mapsto V^{\eta ,\lambda }\left(q,\pi \right)$ (see Lemma 3.4). This together with Lemma 3.5 will imply the desired properties of Φ in order to apply the Kakutani-Fan’s fixed-point theorem.

We have the following result regarding the joint continuity of $\left(q,\pi \right)\mapsto V^{\eta ,\lambda }\left(q,\pi \right)$.

Lemma 3.4 *Let* Assumption 2.1 *hold and* η,μ≥0*. Then the map* $\left(q,\pi \right)\mapsto V^{\eta ,\lambda }\left(q,\pi \right)$ *is continuous for* $\left(q,\pi \right)\in P\left(\Theta \right)\times A^{r}$*.*

Proof Take $\left(q^{n},\pi^{n}\right),\left(q,\pi \right)\in P\left(\Theta \right)\times A^{r}$ with $\left(q^{n},\pi^{n}\right)\to \left(q,\pi \right)$. We have

(19) $$\left|V^{\eta ,\lambda }\left(q^{n},\pi^{n}\right)-V^{\eta ,\lambda }\left(q,\pi \right)\right|\leq \left|V^{\eta ,\lambda }\left(q^{n},\pi^{n}\right)-V^{\eta ,\lambda }\left(q^{n},\pi \right)\right|+\left|V^{\eta ,\lambda }\left(q^{n},\pi \right)-V^{\eta ,\lambda }\left(q,\pi \right)\right|.$$

First let us consider the term $|V^{\eta ,\lambda }\left(q^{n},\pi^{n}\right)-V^{\eta ,\lambda }\left(q^{n},\pi \right)|$. Let ε>0. Choose $T_{0}$ such that

$$\left(2K{\tilde{C}}^{2}+\lambda C\right)\sum_{t=T_{0}}^{\infty }\delta^{t}<\varepsilon ,$$

where $C:=\max_{u\in \Delta }\Gamma \left(u\right)$. Then we choose N∈N such that for any n>N and $t\leq T_{0}$,

$$\begin{array}{ccc} & & sup_{k\in K}∥\pi_{t}^{n}\left(k\right)\left(\cdot \right)-\pi_{t}{\left(k\right)\left(\cdot \right)∥}_{\infty }+{∥\Gamma \left(\pi_{t}^{n}\left(\cdot \right)\right)-\Gamma \left(\pi_{t}\left(\cdot \right)\right)∥}_{\infty }<\tilde{\varepsilon }\\ & & :=\frac{\varepsilon }{K^{T_{0}}{\tilde{C}}^{2}T_{0}^{2}+\lambda \left(T_{0}+CT_{0}^{2}K^{T_{0}}\right)}. \end{array}$$

Then along any path of $x\in \Omega^{X}$ (or $x_{T_{0}-1}$),

$$\left|P_{x}^{\pi^{n}}\left(A_{T_{0}-1}=a_{T_{0}-1}\right)-P_{x}^{\pi }\left(A_{T_{0}-1}=a_{T_{0}-1}\right)\right|\leq T_{0}\tilde{\varepsilon },\;\forall \;a_{T_{0}-1}\in K^{T_{0}}.$$

As a result, for any θ∈Θ,

$$\begin{array}{cc} & \left|E^{\theta ,\pi^{n}}\left[\sum_{t=0}^{T_{0}-1}\delta^{t}\left(X_{t}\left(A_{t}\right)+\lambda \Gamma \left(\pi_{t}^{n}\right)\right)\right]-E^{\theta ,\pi }\left[\sum_{t=0}^{T_{0}-1}\delta^{t}\left(X_{t}\left(A_{t}\right)+\lambda \Gamma \left(\pi_{t}\right)\right)\right]\right|\\ & =\left|E^{\theta }\left[E^{\theta ,\pi^{n}}\left[\sum_{t=0}^{T_{0}-1}\delta^{t}\left(X_{t}\left(A_{t}\right)+\lambda \Gamma \left(\pi_{t}^{n}\right)\right)|X\right]\right]-E^{\theta }\left[E^{\theta ,\pi }\left[\sum_{t=0}^{T_{0}-1}\delta^{t}\left(X_{t}\left(A_{t}\right)+\lambda \Gamma \left(\pi_{t}\right)\right)|X\right]\right]\right|\\ & \leq E^{\theta }\left|E^{\theta ,\pi^{n}}\left[\sum_{t=0}^{T_{0}-1}\delta^{t}\left(X_{t}\left(A_{t}\right)+\lambda \Gamma \left(\pi_{t}^{n}\right)\right)|X\right]-E^{\theta ,\pi }\left[\sum_{t=0}^{T_{0}-1}\delta^{t}\left(X_{t}\left(A_{t}\right)+\lambda \Gamma \left(\pi_{t}\right)\right)|X\right]\right|\\ & =E^{\theta }|\sum_{a_{T_{0}-1}\in K^{T_{0}}}\left(\sum_{t=0}^{T_{0}-1}\delta^{t}\left(X_{t}\left(a_{t}\right)+\lambda \Gamma \left(\pi_{t}^{n}\left(h_{t}\left(a,X\right)\right)\right)\right)\right)\cdot P_{X}^{\pi^{n}}\left(A_{T_{0}-1}=a_{T_{0}-1}\right)\\ & \;-\sum_{a_{T_{0}-1}\in K^{T_{0}}}\left(\sum_{t=0}^{T_{0}-1}\delta^{t}\left(X_{t}\left(a_{t}\right)+\lambda \Gamma \left(\pi_{t}\left(h_{t}\left(a,X\right)\right)\right)\right)\right)\cdot P_{X}^{\pi }\left(A_{T_{0}-1}=a_{T_{0}-1}\right)|\\ & \leq E^{\theta }|\sum_{a_{T_{0}-1}\in K^{T_{0}}}\sum_{t=0}^{T_{0}-1}(\left|X_{t}\left(a_{t}\right)\right|\cdot \left|P_{X}^{\pi^{n}}\left(A_{T_{0}-1}=a_{T_{0}-1}\right)-P_{X}^{\pi }\left(A_{T_{0}-1}=a_{T_{0}-1}\right)\right|\\ & \;+\lambda \left|\Gamma \left(\pi_{t}^{n}\left(h_{t}\left(a,X\right)\right)\right)\cdot P_{X}^{\pi^{n}}\left(A_{T_{0}-1}=a_{T_{0}-1}\right)-\Gamma \left(\pi_{t}\left(h_{t}\left(a,X\right)\right)\right)\cdot P_{X}^{\pi }\left(A_{T_{0}-1}=a_{T_{0}-1}\right)\right|)|\\ & \leq K^{T_{0}}{\tilde{C}}^{2}T_{0}^{2}\tilde{\varepsilon }\\ & \;+\lambda E^{\theta }[\sum_{a_{T_{0}-1}\in K^{T_{0}}}\sum_{t=0}^{T_{0}-1}(\left|\Gamma \left(\pi_{t}^{n}\left(h_{t}\left(a,X\right)\right)\right)-\Gamma \left(\pi_{t}\left(h_{t}\left(a,X\right)\right)\right)\right|\cdot P_{X}^{\pi^{n}}\left(A_{T_{0}-1}=a_{T_{0}-1}\right)\\ & \;+|\Gamma \left(\pi_{t}\left(h_{t}\left(a,X\right)\right)\right)|\cdot \left|P_{X}^{\pi^{n}}\left(A_{T_{0}-1}=a_{T_{0}-1}\right)-P_{X}^{\pi }\left(A_{T_{0}-1}=a_{T_{0}-1}\right)\right|)]\\ & \leq K^{T_{0}}{\tilde{C}}^{2}T_{0}^{2}\tilde{\varepsilon }+\lambda \left(T_{0}+CT_{0}^{2}K^{T_{0}}\right)\tilde{\varepsilon }=\varepsilon , \end{array}$$

where for the simplicity of notation we denote $h_{t}\left(a,X\right):=\left(a_{T_{0}-1},X_{T_{0}-1}\left(a_{T_{0}-1}\right)\right)$ in the above. Therefore, for any n>N,

$$\begin{array}{ccc} & & |E^{\theta ,\pi^{n}}\left[\sum_{t=0}^{\infty }\delta^{t}\left(X_{t}\left(A_{t}\right)+\lambda \Gamma \left(\pi_{t}^{n}\right)\right)\right]\\ & & -E^{\theta ,\pi }\left[\sum_{t=0}^{\infty }\delta^{t}\left(X_{t}\left(A_{t}\right)+\lambda \Gamma \left(\pi_{t}\right)\right)\right]|\leq 3\varepsilon ,\;\forall \;\theta \in \Theta , \end{array}$$

and thus

$$|V^{\eta ,\lambda }\left(q^{n},\pi^{n}\right)-V^{\eta ,\lambda }\left(q^{n},\pi \right)|\leq 3\varepsilon .$$

That is, $|V^{\eta ,\lambda }\left(q^{n},\pi^{n}\right)-V^{\eta ,\lambda }\left(q^{n},\pi \right)|\to 0$ as n→∞.

Next, we consider $|V^{\eta ,\lambda }\left(q^{n},\pi \right)-V^{\eta ,\lambda }\left(q,\pi \right)|$. Let $\theta^{n},\theta \in \Theta$ with $\theta^{n}\to \theta$. For each t∈N, similar to (16) we have that

$$E^{\theta_{n},\pi }\left[X_{t}\left(A_{t}\right)\right]=\sum_{k=1}^{K}E^{\theta_{n}}\left[X_{t}\left(k\right)\right]\cdot P^{\theta_{n},\pi }\left(A_{t}=k\right).$$

We have that

$$\begin{array}{cc} & P^{\theta_{n},\pi }\left(A_{t}=k\right)=\sum_{a_{t-1}\in K^{t}}E^{\theta_{n},\pi }\left[E^{\theta_{n},\pi }\left[1_{\left\{A_{t-1}=a_{t-1},A_{t}=k\right\}}\;|\;X\right]\right]\\ & =\sum_{a_{t-1}\in K^{t}}E^{\theta_{n}}\left[\pi_{0}\left(a_{0}\right)\cdots \pi_{t-1}\left(a_{t-1}\right)\left(a_{t-2},X_{t-2}\left(a_{t-2}\right)\right)\cdot \pi_{t}\left(k\right)\left(a_{t-1},X_{t-1}\left(a_{t-1}\right)\right)\right]\\ & =\sum_{a_{t-1}\in K^{t}}E^{*}[f_{a_{0}}^{\theta_{n}}\left(X_{0}\left(a_{0}\right)\right)\cdots f_{a_{t-1}}^{\theta_{n}}\left(X_{t-1}\left(a_{t-1}\right)\right)\\ & \cdot \pi_{0}\left(a_{0}\right)\cdots \pi_{t-1}\left(a_{t-1}\right)\left(a_{t-2},X_{t-2}\left(a_{t-2}\right)\right)\cdot \pi_{t}\left(k\right)\left(a_{t-1},X_{t-1}\left(a_{t-1}\right)\right)]\\ & \to \sum_{a_{t-1}\in K^{t}}E^{*}[f_{a_{0}}^{\theta }\left(X_{0}\left(a_{0}\right)\right)\cdots f_{a_{t-1}}^{\theta }\left(X_{t-1}\left(a_{t-1}\right)\right)\\ & \cdot \pi_{0}\left(a_{0}\right)\cdots \pi_{t-1}\left(a_{t-1}\right)\left(a_{t-2},X_{t-2}\left(a_{t-2}\right)\right)\cdot \pi_{t}\left(k\right)\left(a_{t-1},X_{t-1}\left(a_{t-1}\right)\right)]\\ & =P^{\theta ,\pi }\left(A_{t}=k\right). \end{array}$$

As a result, we have that

(20) $$E^{\theta_{n},\pi }\left[X_{t}\left(A_{t}\right)\right]\to E^{\theta ,\pi }\left[X_{t}\left(A_{t}\right)\right],\;n\to \infty .$$

Similarly, for each t∈N we have that

(21) $$\begin{array}{cc} & E^{\theta^{n},\pi }\left[\Gamma (\pi_{t}\left(H_{t-1}\right))\right]=\sum_{a_{t-1}\in K^{t}}E^{\theta_{n},\pi }\left[E^{\theta_{n},\pi }\left[\Gamma \left(\pi_{t}\left(H_{t-1}\right)\right)\cdot 1_{\left\{A_{t-1}=a_{t-1}\right\}}\;|\;X\right]\right]\\ & =\sum_{a_{t-1}\in K^{t}}E^{\theta_{n},\pi }\left[E^{\theta_{n},\pi }\left[\Gamma \left(\pi_{t}\left(a_{t-1},X_{t-1}\left(a_{t-1}\right)\right)\right)\cdot 1_{\left\{A_{t-1}=a_{t-1}\right\}}\;|\;X\right]\right]\\ & =\sum_{a_{t-1}\in K^{t}}E^{\theta_{n},\pi }\left[\Gamma \left(\pi_{t}\left(a_{t-1},X_{t-1}\left(a_{t-1}\right)\right)\right)\cdot E^{\theta_{n},\pi }\left[1_{\left\{A_{t-1}=a_{t-1}\right\}}\;|\;X\right]\right]\\ & =\sum_{a_{t-1}\in K^{t}}E^{\theta_{n}}\left[\Gamma \left(\pi_{t}\left(a_{t-1},X_{t-1}\left(a_{t-1}\right)\right)\right)\cdot \pi_{0}\left(a_{0}\right)\cdots \pi_{t-1}\left(a_{t-1}\right)\left(a_{t-2},X_{t-2}\left(a_{t-2}\right)\right)\right]\\ & \to \sum_{a_{t-1}\in K^{t}}E^{\theta }\left[\Gamma \left(\pi_{t}\left(a_{t-1},X_{t-1}\left(a_{t-1}\right)\right)\right)\cdot \pi_{0}\left(a_{0}\right)\cdots \pi_{t-1}\left(a_{t-1}\right)\left(a_{t-2},X_{t-2}\left(a_{t-2}\right)\right)\right]\\ & =E^{\theta ,\pi }\left[\Gamma (\pi_{t}\left(H_{t-1}\right))\right]. \end{array}$$

From (20), (21) and using a truncation argument for the time horizon, we can show that for fixed $\pi \in A^{r}$ the map $\theta \mapsto V^{0,\lambda }\left(\theta ,\pi \right)$ is continuous. Then the map

$$\tilde{q}\mapsto V^{\eta ,\lambda }\left(\tilde{q},\pi \right)=\int_{\Theta }V^{0,\lambda }\left(\theta ,\pi \right)\tilde{q}\left(d\theta \right)+\eta \psi \left(\tilde{q}\right)$$

is continuous. Therefore, in (19) the term $|V^{\eta ,\lambda }\left(q^{n},\pi \right)-V^{\eta ,\lambda }\left(q,\pi \right)|\to 0$. □

Under Assumption 2.2(i), w.l.o.g. we adjust $H_{t}$ and define

$$H_{t}:=\left\{\left(a_{0},\cdots ,a_{t},x_{0},\cdots ,x_{t}\right):\;a_{s}\in K,x_{s}\in S_{a_{s}},\;s=1,\cdots ,t\right\}.$$

Consider the problem

(22) $$F\left(q\right):=sup_{\pi \in A^{r}}\int_{\Theta }E^{\theta ,\pi }\left[\sum_{t=0}^{\infty }\delta^{t}\left(X_{t}\left(A_{t}\right)+\lambda \Gamma \left(\pi_{t}\right)\right)\right]q\left(d\theta \right),\;q\in P\left(\Theta \right).$$

This is a Bayesian optimization problem and F(q), q∈P(Θ), satisfies the dynamic programming equation

(23) $$F\left(q\right)=sup_{u\in \Delta }\sum_{k\in K}\left[\int_{\Theta }\int_{S_{k}}\left(z+\delta F(q{|}_{k,z})\right)p_{k}^{\theta }\left(dz\right)q\left(d\theta \right)\cdot u_{k}-\lambda u_{k}\ln u_{k}\right].$$

Here ${q|}_{k,z}$ is the posterior distribution of θ given the outcome of the k-th arm is z, i.e.,

$${q|}_{k,z}\left(d\theta \right)=\frac{f_{k}^{\theta }\left(z\right)q\left(d\theta \right)}{\int_{\Theta }f_{k}^{\tilde{\theta }}\left(z\right)q\left(d\tilde{\theta }\right)}.$$

The optimizer $\hat{u}=\left({\hat{u}}_{1},\cdots ,{\hat{u}}_{K}\right)\in \Delta$ for the supremum in (23) is unique and given by

$${\hat{u}}_{k}\left(q\right)=\frac{E\left[(\right]\frac{1}{\lambda }\left(\int_{\Theta }\int_{S_{k}}\left(z+\delta F(q{|}_{k,z})\right)p_{k}^{\theta }\left(dz\right)q\left(d\theta \right)\right)}{\sum_{i=1}^{K}E\left[(\right]\frac{1}{\lambda }\left(\int_{\Theta }\int_{S_{i}}\left(z+\delta F(q{|}_{i,z})\right)p_{i}^{\theta }\left(dz\right)q\left(d\theta \right)\right)}.$$

Therefore, the optimal solution $\hat{\pi }\in A^{r}$ for (22) is given by

(24) $${\hat{\pi }}_{t}\left(k\right)\left(h_{t-1}\right)={\hat{u}}_{k}\left(q{|}_{h_{t-1}}\right)=\frac{E\left[(\right]\frac{1}{\lambda }\left(\int_{\Theta }\int_{S_{k}}\left(z+\delta F(q{|}_{a_{t-1},k,x_{t-1},z})\right)p_{k}^{\theta }\left(dz\right)q{|}_{a_{t-1},x_{t-1}}\left(d\theta \right)\right)}{\sum_{i=1}^{K}E\left[(\right]\frac{1}{\lambda }\left(\int_{\Theta }\int_{S_{i}}\left(z+\delta F(q{|}_{a_{t-1},i,x_{t-1},z})\right)p_{i}^{\theta }\left(dz\right)q{|}_{a_{t-1},x_{t-1}}\left(d\theta \right)\right)},$$

where for $h_{t-1}=\left(a_{0},\cdots ,a_{t-1},x_{0},\cdots ,x_{t-1}\right)\in H_{t-1}$,

$${q|}_{h_{t-1}}\left(d\theta \right)=\frac{\Pi_{s=0}^{t-1}\;f_{a_{s}}^{\theta }\left(x_{s}\right)q\left(d\theta \right)}{\int_{\Theta }\Pi_{s=0}^{t-1}\;f_{a_{s}}^{\tilde{\theta }}\left(x_{s}\right)q\left(d\tilde{\theta }\right)}.$$

Note that the optimal solution for (22) is unique. Indeed, suppose there exists another optimal solution $\tilde{\pi }\in A^{r}$ that is different from $\hat{\pi }$. Then there exists $t^{*}\in N$, $a_{t^{*}-1}\in K^{t^{*}}$ and $B^{*}\subset S_{a_{0}}\times \cdots \times S_{a_{t^{*}-1}}$, such that

$$p_{a_{0}^{*}}^{*}\times \cdots \times p_{a_{t^{*}-1}}^{*}\left(B^{*}\right)>0\;⟺\;\int_{\Theta }p_{a_{0}^{*}}^{\theta }\times \cdots \times p_{a_{t^{*}-1}}^{\theta }\left(B^{*}\right)\;q\left(d\theta \right)>0,$$

and

(25) $${\tilde{\pi }}_{t^{*}}\left(a_{t^{*}-1},x_{t^{*}-1}\right)\neq {\hat{\pi }}_{t^{*}}\left(a_{t^{*}-1},x_{t^{*}-1}\right),\;\forall \;x_{t^{*}-1}\in B^{*}.$$

We choose the minimum of such $t^{*}$. Then for $t<t^{*}$ we have

$${\tilde{\pi }}_{t}\left(k\right)={\hat{\pi }}_{t}\left(k\right)>0.$$

As a result,

(26) $$\int_{\Theta }P^{\theta ,\tilde{\pi }}\left(H_{t^{*}-1}\in B\right)\;q\left(d\theta \right)=\int_{\Theta }P^{\theta ,\hat{\pi }}\left(H_{t^{*}-1}\in B\right)\;q\left(d\theta \right),\;\forall \;B\subset H_{t^{*}-1},$$

and

(27) $$\begin{array}{cc} & \int_{\Theta }P^{\theta ,\tilde{\pi }}\left(A_{t^{*}-1}=a_{t^{*}-1},X_{t^{*}-1}\left(a_{t^{*}-1}\right)\in B^{*}\right)\;q\left(d\theta \right)\\ & =\int_{\Theta }P^{\theta ,\hat{\pi }}\left(A_{t^{*}-1}=a_{t^{*}-1},X_{t^{*}-1}\left(a_{t^{*}-1}\right)\in B^{*}\right)\;q\left(d\theta \right)>0. \end{array}$$

Denote ${\bar{E}}^{\pi }\left[\cdot \right]:=\int_{\Theta }E^{\theta ,\pi }\left[\cdot \right]q\left(d\theta \right)$ for $\pi =\hat{\pi },\tilde{\pi }$. By (25), for $A_{t^{*}-1}=a_{t^{*}-1}$ and $X_{t^{*}-1}\left(a_{t^{*}-1}\right)\in B^{*}$,

$$\begin{array}{ccc} & & \delta^{t^{*}}F\left({q|}_{H_{t^{*}-1}}\right)\\ & & =\;{\bar{E}}^{\hat{\pi }}\left[\sum_{t=t^{*}}^{\infty }\delta^{t}\left(X_{t}\left(A_{t}\right)+\lambda \Gamma \left(\pi_{t}\right)\right)\;|\;H_{t^{*}-1}\right]\\ & & \;>{\bar{E}}^{\tilde{\pi }}\left[\sum_{t=t^{*}}^{\infty }\delta^{t}\left(X_{t}\left(A_{t}\right)+\lambda \Gamma \left(\pi_{t}\right)\right)\;|\;H_{t^{*}-1}\right]. \end{array}$$

Then

$$\begin{array}{cc} & {\bar{E}}^{\tilde{\pi }}\left[\sum_{t=0}^{\infty }\delta^{t}\left(X_{t}\left(A_{t}\right)+\lambda \Gamma \left(\pi_{t}\right)\right)\right]\\ & ={\bar{E}}^{\tilde{\pi }}\left[\sum_{t=0}^{t^{*}-1}\delta^{t}\left(X_{t}\left(A_{t}\right)+\lambda \Gamma \left(\pi_{t}\right)\right)+{\bar{E}}^{\tilde{\pi }}\left[\sum_{t=t^{*}}^{\infty }\delta^{t}\left(X_{t}\left(A_{t}\right)+\lambda \Gamma \left(\pi_{t}\right)\right)\;|\;H_{t^{*}-1}\right]\right]\\ & <{\bar{E}}^{\tilde{\pi }}\left[\sum_{t=0}^{t^{*}-1}\delta^{t}\left(X_{t}\left(A_{t}\right)+\lambda \Gamma \left(\pi_{t}\right)\right)+\delta^{t^{*}}F\left({q|}_{H_{t^{*}-1}}\right)\right]\\ & ={\bar{E}}^{\hat{\pi }}\left[\sum_{t=0}^{t^{*}-1}\delta^{t}\left(X_{t}\left(A_{t}\right)+\lambda \Gamma \left(\pi_{t}\right)\right)+\delta^{t^{*}}F\left({q|}_{H_{t^{*}-1}}\right)\right]=F\left(q\right), \end{array}$$

where the third line follows from (27) and the fourth line follows from (26). This contradicts $\tilde{\pi }$ being optimal for (22).

Lemma 3.5 *Let* Assumptions 2.1 *and* 2.2 *hold. Consider* $\hat{\pi }\in A^{r}$ *given in* (24)*. For each* t∈N*, there exists a modulus of continuity function* $\rho_{t}\left(\cdot \right)$ *independent of* q∈P(θ)*, such that*

(28) $$|{\hat{\pi }}_{t}\left(k\right)\left(a_{t-1},x_{t-1}\right)-{\hat{\pi }}_{t}\left(k\right)\left(a_{t-1},y_{t-1}\right)|\leq \rho_{t}(∥x_{t-1},y_{t-1}{∥}_{\infty }),$$

*for any* $k\in K,\;a_{t-1}\in K^{t},\;x_{t-1},y_{t-1}\in S_{a_{0}}\times \cdots \times S_{a_{t-1}}$*.*

Proof Let t∈N. Throughout the proof, C>0 will be a constant and $\tilde{\rho }\left(\cdot \right)$ will be a modulus of continuity function, and they may change from line to line but are always independent of q∈P(Θ).

Take $a_{t-1}\in K^{t}$ and $x_{t-1},y_{t-1}\in S_{a_{0}}\times \cdots \times S_{a_{t-1}}$. We have that

$$\begin{array}{cc} & \left|{q|}_{a_{t-1},x_{t-1}}{\left(d\theta \right)-q|}_{a_{t-1},y_{t-1}}\left(d\theta \right)\right|=\left|\frac{\Pi_{s=0}^{t-1}\;f_{a_{s}}^{\theta }\left(x_{s}\right)q\left(d\theta \right)}{\int_{\Theta }\Pi_{s=0}^{t-1}\;f_{a_{s}}^{\tilde{\theta }}\left(x_{s}\right)q\left(d\tilde{\theta }\right)}-\frac{\Pi_{s=0}^{t-1}\;f_{a_{s}}^{\theta }\left(y_{s}\right)q\left(d\theta \right)}{\int_{\Theta }\Pi_{s=0}^{t-1}\;f_{a_{s}}^{\tilde{\theta }}\left(y_{s}\right)q\left(d\tilde{\theta }\right)}\right|\\ & =|\frac{\Pi_{s=0}^{t-1}\;f_{a_{s}}^{\theta }\left(x_{s}\right)q\left(d\theta \right)\cdot \int_{\Theta }\Pi_{s=0}^{t-1}\;f_{a_{s}}^{\tilde{\theta }}\left(y_{s}\right)q\left(d\tilde{\theta }\right)-\Pi_{s=0}^{t-1}\;f_{a_{s}}^{\theta }\left(x_{s}\right)q\left(d\theta \right)\cdot \int_{\Theta }\Pi_{s=0}^{t-1}\;f_{a_{s}}^{\tilde{\theta }}\left(x_{s}\right)q\left(d\tilde{\theta }\right)}{\int_{\Theta }\Pi_{s=0}^{t-1}\;f_{a_{s}}^{\tilde{\theta }}\left(x_{s}\right)q\left(d\tilde{\theta }\right)\cdot \int_{\Theta }\Pi_{s=0}^{t-1}\;f_{a_{s}}^{\tilde{\theta }}\left(y_{s}\right)q\left(d\tilde{\theta }\right)}\\ & \;+\frac{\Pi_{s=0}^{t-1}\;f_{a_{s}}^{\theta }\left(x_{s}\right)q\left(d\theta \right)\cdot \int_{\Theta }\Pi_{s=0}^{t-1}\;f_{a_{s}}^{\tilde{\theta }}\left(x_{s}\right)q\left(d\tilde{\theta }\right)-\Pi_{s=0}^{t-1}\;f_{a_{s}}^{\theta }\left(y_{s}\right)q\left(d\theta \right)\cdot \int_{\Theta }\Pi_{s=0}^{t-1}\;f_{a_{s}}^{\tilde{\theta }}\left(x_{s}\right)q\left(d\tilde{\theta }\right)}{\int_{\Theta }\Pi_{s=0}^{t-1}\;f_{a_{s}}^{\tilde{\theta }}\left(x_{s}\right)q\left(d\tilde{\theta }\right)\cdot \int_{\Theta }\Pi_{s=0}^{t-1}\;f_{a_{s}}^{\tilde{\theta }}\left(y_{s}\right)q\left(d\tilde{\theta }\right)}|\\ & \leq C\cdot \Pi_{s=0}^{t-1}\;f_{a_{s}}^{\theta }\left(x_{s}\right)q\left(d\theta \right)\cdot \int_{\Theta }\left|\Pi_{s=0}^{t-1}\;f_{a_{s}}^{\tilde{\theta }}\left(y_{s}\right)-\Pi_{s=0}^{t-1}\;f_{a_{s}}^{\tilde{\theta }}\left(x_{s}\right)\right|q\left(d\tilde{\theta }\right)\\ & \;+C\cdot \int_{\Theta }\Pi_{s=0}^{t-1}\;f_{a_{s}}^{\tilde{\theta }}\left(x_{s}\right)q\left(d\tilde{\theta }\right)\cdot \left|\Pi_{s=0}^{t-1}\;f_{a_{s}}^{\theta }\left(x_{s}\right)-\Pi_{s=0}^{t-1}\;f_{a_{s}}^{\theta }\left(y_{s}\right)\right|q\left(d\theta \right)\\ & \leq \tilde{\rho }(∥x_{t-1}-y_{t-1}{∥}_{\infty })q\left(d\theta \right). \end{array}$$

As a result, for k∈K and $z\in S_{k}$,

$$\begin{array}{cc} & |F\left(q{|}_{a_{t-1},i,x_{t-1},z}\right)-F\left(q{|}_{a_{t-1},i,y_{t-1},z}\right)|\\ & =|sup_{\pi }\int_{\Theta }E^{\theta ,\pi }\left[\sum_{t=0}^{T}\delta^{t}\left(X_{t}\left(A_{t}\right)+\lambda \Gamma \left(\pi_{t}\right)\right)\right]{q|}_{a_{t-1},i,x_{t-1},z}\left(d\theta \right)\\ & -sup_{\pi }\int_{\Theta }E^{\theta ,\pi }\left[\sum_{t=0}^{T}\delta^{t}\left(X_{t}\left(A_{t}\right)+\lambda \Gamma \left(\pi_{t}\right)\right)\right]q{|}_{a_{t-1},i,y_{t-1},z}\left(d\theta \right)|\\ & \leq sup_{\pi }\int_{\Theta }\left|E^{\theta ,\pi }\left[\sum_{t=0}^{T}\delta^{t}\left(X_{t}\left(A_{t}\right)+\lambda \Gamma \left(\pi_{t}\right)\right)\right]\right|\cdot \left|{q|}_{a_{t-1},i,x_{t-1},z}{\left(d\theta \right)-q|}_{a_{t-1},i,x_{t-1},z}\left(d\theta \right)\right|\\ & \leq C\cdot \int_{\Theta }\left|{q|}_{a_{t-1},i,x_{t-1},z}{\left(d\theta \right)-q|}_{a_{t-1},i,x_{t-1},z}\left(d\theta \right)\right|\\ & \leq C\cdot \int_{\Theta }\tilde{\rho }(∥\left(x_{t-1},z\right)-\left(y_{t-1},z\right){∥}_{\infty })q\left(d\theta \right).\\ & \leq \tilde{\rho }(∥x_{t-1}-y_{t-1}{∥}_{\infty }). \end{array}$$

Then

$$\begin{array}{cc} & |\int_{\Theta }\int_{S_{k}}\left(z+\delta F(q{|}_{a_{t-1},k,x_{t-1},z})\right)p_{k}^{\theta }\left(dz\right)q{|}_{a_{t-1},x_{t-1}}\left(d\theta \right))\\ & \;-\int_{\Theta }\int_{S_{k}}\left(z+\delta F(q{|}_{a_{t-1},k,y_{t-1},z})\right)p_{k}^{\theta }{\left(dz\right)q|}_{a_{t-1},y_{t-1}}\left(d\theta \right))|\\ & \leq \left|\int_{\Theta }\int_{S_{k}}zp_{k}^{\theta }{\left(dz\right)q|}_{a_{t-1},x_{t-1}}\left(d\theta \right))-\int_{\Theta }\int_{S_{k}}zp_{k}^{\theta }\left(dz\right)q{|}_{a_{t-1},y_{t-1}}\left(d\theta \right))\right|\\ & \;+|\int_{\Theta }\int_{S_{k}}F\left(q{|}_{a_{t-1},k,x_{t-1},z}\right)p_{k}^{\theta }\left(dz\right)q{|}_{a_{t-1},x_{t-1}}\left(d\theta \right))\\ & \;-\int_{\Theta }\int_{S_{k}}F\left(q{|}_{a_{t-1},k,y_{t-1},z}\right)p_{k}^{\theta }\left(dz\right)q{|}_{a_{t-1},y_{t-1}}\left(d\theta \right))|\\ & \leq \int_{\Theta }\left|\int_{S_{k}}zp_{k}^{\theta }\left(dz\right)\right|\cdot \left|{q|}_{a_{t-1},x_{t-1}}{\left(d\theta \right))-q|}_{a_{t-1},y_{t-1}}\left(d\theta \right))\right|\\ & \;+|\int_{\Theta }\int_{S_{k}}F\left(q{|}_{a_{t-1},k,x_{t-1},z}\right)p_{k}^{\theta }\left(dz\right)q{|}_{a_{t-1},x_{t-1}}\left(d\theta \right))\\ & \;-\int_{\Theta }\int_{S_{k}}F\left(q{|}_{a_{t-1},k,x_{t-1},z}\right)p_{k}^{\theta }\left(dz\right)q{|}_{a_{t-1},y_{t-1}}\left(d\theta \right))|\\ & \;+|\int_{\Theta }\int_{S_{k}}F\left(q{|}_{a_{t-1},k,x_{t-1},z}\right)p_{k}^{\theta }\left(dz\right)q{|}_{a_{t-1},y_{t-1}}\left(d\theta \right))\\ & \;-\int_{\Theta }\int_{S_{k}}F\left(q{|}_{a_{t-1},k,y_{t-1},z}\right)p_{k}^{\theta }\left(dz\right)q{|}_{a_{t-1},y_{t-1}}\left(d\theta \right))|\\ & \leq C\cdot \int_{\Theta }\left|{q|}_{a_{t-1},x_{t-1}}{\left(d\theta \right))-q|}_{a_{t-1},y_{t-1}}\left(d\theta \right))\right|\\ & \;+\int_{\Theta }\left|\int_{S_{k}}F\left(q{|}_{a_{t-1},k,x_{t-1},z}\right)p_{k}^{\theta }\left(dz\right)\right|\cdot \left|{q|}_{a_{t-1},x_{t-1}}{\left(d\theta \right))-q|}_{a_{t-1},y_{t-1}}\left(d\theta \right))\right|\\ & \;+\left|\int_{\Theta }\int_{S_{k}}\left|F\left(q{|}_{a_{t-1},k,x_{t-1},z}\right)-F\left(q{|}_{a_{t-1},k,y_{t-1},z}\right)\right|p_{k}^{\theta }\left(dz\right)q{|}_{a_{t-1},y_{t-1}}\left(d\theta \right))\right|\\ & \leq \tilde{\rho }(∥x_{t-1}-y_{t-1}{∥}_{\infty })+C\cdot \int_{\Theta }\left|{q|}_{a_{t-1},x_{t-1}}{\left(d\theta \right))-q|}_{a_{t-1},y_{t-1}}\left(d\theta \right))\right|\\ & \;+\left|\int_{\Theta }\int_{S_{k}}\tilde{\rho }(∥x_{t-1}-y_{t-1}{∥}_{\infty })p_{k}^{\theta }\left(dz\right)q{|}_{a_{t-1},y_{t-1}}\left(d\theta \right))\right|\\ & \leq \tilde{\rho }(∥x_{t-1}-y_{t-1}{∥}_{\infty }). \end{array}$$

Since the term

$$\int_{\Theta }\int_{S_{k}}\left(z+\delta F(q{|}_{a_{t-1},k,x_{t-1},z})\right)p_{k}^{\theta }\left(dz\right)q{|}_{a_{t-1},x_{t-1}}\left(d\theta \right))$$

is bounded uniformly in k∈K,q∈P(Θ) and $\left(a_{t-1},x_{t-1}\right)\in H_{t-1}$, the result follows. □

We are ready for the proof of Theorem 2.2.

Proof of Theorem 2.2 Given $\rho_{t}$ for t∈N from (28), define ${\bar{A}}^{r}\subset A^{r}$ by

(29) $${\bar{A}}^{r}:=\left\{\pi \in A^{r}:\;\pi_{t}\;\text{satisfies}\;\text{(28)},\;\forall \;t\in N\right\}.$$

Recall $d_{A^{r}}$ defined in (12). The set ${\bar{A}}^{r}$ is a compact separable metric space under $d_{A^{r}}$. Let η≥0 and λ>0. Consider the set valued mapping $\Phi :\;P\left(\Theta \right)\times {\bar{A}}^{r}\mapsto 2^{P\left(\Theta \right)\times {\bar{A}}^{r}}$,

(30) $$\Phi \left(q,\pi \right):=\left\{\left(\tilde{q},\tilde{\pi }\right):\;\tilde{q}\in \text{arg}\max_{\hat{q}\in P\left(\Theta \right)}V^{\eta ,\lambda }\left(\hat{q},\pi \right),\;\tilde{\pi }\in \text{arg}\max_{\hat{\pi }\in {\bar{A}}^{r}}V^{\eta ,\lambda }\left(q,\hat{\pi }\right)\right\}.$$

By Lemmas 3.4 and 3.5, Φ(·) is nonempty. For fixed $\pi \in {\bar{A}}^{r}$, $\text{arg}\max_{\hat{q}\in P\left(\Theta \right)}V^{\eta ,\lambda }\left(\hat{q},\pi \right)$ is convex and compact, and for fixed q∈P(Θ), $\text{arg}\max_{\hat{\pi }\in {\bar{A}}^{r}}V^{\eta ,\lambda }\left(q,\hat{\pi }\right)$ is a singleton. Moreover, Lemma 3.4 implies Φ is upper-semicontinuous. By Kakutani-Fan’s fixed point theorem, there exists $\left(q^{*},\pi^{*}\right)\in P\left(\Theta \right)\times {\bar{A}}^{r}$ such that for any $\left(q,\pi \right)\in P\left(\Theta \right)\times {\bar{A}}^{r}$,

(31) $$V^{\eta ,\lambda }\left(q,\pi^{*}\right)\leq V^{\eta ,\lambda }\left(q^{*},\pi^{*}\right)\leq V^{\eta ,\lambda }\left(q^{*},\pi \right).$$

Note for any q∈P(Θ), the optimizer for $inf_{\pi \in A^{r}}V^{\eta ,\lambda }\left(q,\pi \right)$ is uniquely given by (24) which is in ${\bar{A}}^{r}$. Consequently, (31) holds for any $\left(q,\pi \right)\in P\left(\Theta \right)\times A^{r}$, i.e., $\left(q^{*},\pi^{*}\right)$ is a saddle point for $V^{\eta ,\lambda }$ on $P\left(\Theta \right)\times A^{r}$, and $\pi^{*}$ is the unique optimizer for $inf_{\pi \in A^{r}}V^{\eta ,\lambda }\left(q^{*},\pi \right)$.

Now suppose η>0. Let $\left(\bar{q},\bar{\pi }\right)$ be another saddle point. Since $q\mapsto inf_{\pi }V^{\eta ,\lambda }\left(q,\pi \right)$ is strictly concave, we have $\bar{q}=q^{*}$. As both $\bar{\pi }$ and $\pi^{*}$ are optimizers for the problem $inf_{\pi \in A^{r}}V^{\eta ,\lambda }\left(q^{*},\pi \right)$, we have $\bar{\pi }=\pi^{*}$ since its optimal solution is unique. That is, $\left(q^{*},\pi^{*}\right)$ is the unique saddle point. □

Proof of Corollary 2.1 By (11), for any $\left(q,\pi \right)\in P\left(\Theta \right)\times A^{r}$,

(32) $$\begin{array}{cc} & V\left(q,\pi^{*}\right)-\eta \psi \left(q\right)-\lambda \int_{\Theta }E^{\theta ,\pi^{*}}\left[\sum_{t=0}^{T}\delta^{t}\Gamma \left(\pi_{t}\right)\right]q\left(d\theta \right)\\ & \leq V\left(q^{*},\pi^{*}\right)-\eta \psi \left(q^{*}\right)-\lambda \int_{\Theta }E^{\theta ,\pi^{*}}\left[\sum_{t=0}^{T}\delta^{t}\Gamma \left(\pi_{t}\right)\right]q^{*}\left(d\theta \right) \end{array}$$

(33) $$\begin{array}{cc} & \leq V\left(q^{*},\pi \right)-\eta \psi \left(q^{*}\right)-\lambda \int_{\Theta }E^{\theta ,\pi }\left[\sum_{t=0}^{T}\delta^{t}\Gamma \left(\pi_{t}\right)\right]q^{*}\left(d\theta \right). \end{array}$$

By (32),

$$V\left(q,\pi^{*}\right)-\left(C_{1}\eta +C_{2}\lambda \right)\leq V\left(q^{*},\pi^{*}\right),\;\forall \;q\in P\left(\Theta \right),$$

and by (33)

$$V\left(q^{*},\pi^{*}\right)-\left(C_{1}\eta +C_{2}\lambda \right)\leq V\left(q^{*},\pi \right),\;\forall \;\pi \in A^{r}.$$

Hence, $\left(q^{*},\pi^{*}\right)$ is a $\left(C_{1}\eta +C_{2}\lambda \right)$-saddle point. Then

$$\begin{array}{cc} & \underset{q\in P(\Theta )}{\sup}V\left(q,\pi^{*}\right)\leq V\left(q^{*},\pi^{*}\right)+\left(C_{1}\eta +C_{2}\lambda \right)\\ & \;\leq \underset{\pi \in A^{r}}{\inf}V\left(q^{*},\pi \right)+2\left(C_{1}\eta +C_{2}\lambda \right)\leq \underset{\pi \in A^{r}}{\inf}\underset{q\in P(\Theta )}{\sup}V\left(q,\pi \right)+2\left(C_{1}\eta +C_{2}\lambda \right). \end{array}$$

 □

Proof of Proposition 2.2 For any $\left(q,\pi \right)\in P\left(\Theta \right)\times A^{r}$, we have that

$$\begin{array}{cc} & V\left(q,\pi^{n}\right)-\eta^{n}\psi \left(q\right)-\lambda^{n}\int_{\Theta }E^{\theta ,\pi^{n}}\left[\sum_{t=0}^{T}\delta^{t}\Gamma \left(\pi_{t}\right)\right]q\left(d\theta \right)\\ & \leq V\left(q^{n},\pi^{n}\right)-\eta^{n}\psi \left(q^{n}\right)-\lambda^{n}\int_{\Theta }E^{\theta ,\pi^{n}}\left[\sum_{t=0}^{T}\delta^{t}\Gamma \left(\pi_{t}\right)\right]q^{n}\left(d\theta \right)\\ & \leq V\left(q^{n},\pi \right)-\eta^{n}\psi \left(q^{n}\right)-\lambda^{n}\int_{\Theta }E^{\theta ,\pi }\left[\sum_{t=0}^{T}\delta^{t}\Gamma \left(\pi_{t}\right)\right]q^{n}\left(d\theta \right). \end{array}$$

Sending n→∞ and applying Lemma3.4, we get that

$$V\left(q,\hat{\pi }\right)\leq V\left(\hat{q},\hat{\pi }\right)\leq V\left(\hat{q},\pi \right),\;\forall \;\left(q,\pi \right)\in P\left(\Theta \right)\times A^{r}.$$

 □

## CRediT authorship contribution statement

**Zhou Zhou:** Writing – review & editing, Writing – original draft, Validation, Resources, Project administration, Methodology, Investigation, Formal analysis, Conceptualization.

## Declaration of competing interest

The author is an Editorial Board Member/Editor-in-Chief/Associate Editor/Guest Editor for this journal and was not involved in the editorial review or the decision to publish this article.

## Footnotes

1 The uniqueness is in the sense that, if $\tilde{\pi }$ is another optimizer for $inf_{\pi \in A^{r}}V^{\eta ,\lambda }\left(q^{*},\pi \right)$, then it must hold that ${\tilde{\pi }}_{t}\left(a_{0},\cdots ,a_{t-1},X_{0}\left(a_{0}\right),\cdots ,X_{t-1}\left(a_{t-1}\right)\right)=\pi_{t}^{*}\left(a_{0},\cdots ,a_{t-1},X_{0}\left(a_{0}\right),\cdots ,X_{t-1}\left(a_{t-1}\right)\right),\;p_{a_{0}}^{*}\times \cdots \times p_{a_{t-1}}^{*}\text{-a.s.},$ for any t∈N and $a_{0},\cdots ,a_{t-1}\in K$.
